# A novel lytic phage with potent efficacy against *Bacillus cereus*, its biofilms and spores, and biocontrol potential in food matrices

**DOI:** 10.1016/j.crfs.2026.101603

**Published:** 2026-10-19

**Authors:** Peng Wan, Zhengyuan He, Weiqi Liu, Honghao Huang, Huan Chen, Sihan Lin, Wenguang Xiong, Zhenling Zeng

**Affiliations:** a National Risk Assessment Laboratory for Antimicrobial Resistance of Animal Original Bacteria, College of Veterinary Medicine, South China Agricultural University, Guangzhou, 510642, China; b Guangdong Provincial Key Laboratory of Veterinary Pharmaceutics Development and Safety Evaluation, South China Agricultural University, Guangzhou, 510642, China

**Keywords:** *Bacillus cereus*, Phage, Biocontrol, Food matrices

## Abstract

To address the severe threat posed by *Bacillus cereus* (*B. cereus*) and its spores to food safety, this study isolated and characterized a novel lytic phage, WAH2, using a multidrug-resistant *B. cereus* strain as the host. Morphological observation showed that WAH2 had an icosahedral head and a contractile tail. The phage lysed 7 of 51 (13.7%) *B. cereus* strains covering 3 sequence types. Biological characterization showed that the optimal multiplicity of infection of WAH2 was 0.0001. It remained stable at 4–45 °C, pH 5–11, and 2%–10% NaCl. Its latent period was 30 min, and the burst size was 208 PFU/cell. Genome analysis showed that WAH2 possesses a linear double-stranded DNA genome of 158991 bp with a GC content of 37.96%. It encodes 284 open reading frames and 6 tRNA genes. No antibiotic resistance genes, virulence factors, or lysogeny-related elements were detected. WAH2 was classified as a novel species within the genus *Bastillevirus*, family *Herelleviridae*. *In vitro* assays showed that WAH2 strongly inhibited host bacterial growth (no regrowth within 36 h), significantly reduced biofilm formation and disrupted mature biofilms, and effectively inhibited the outgrowth of germinated spores. Importantly, in food matrices such as milk, rice, and pork, WAH2 significantly reduced or completely eliminated *B. cereus* in a dose- and time-dependent manner at both 25 and 4 °C, with no regrowth observed. In TSB and milk, WAH2 persisted for 120 h without detectable resistant survivors. In summary, WAH2 is a safe, efficient, and promising phage that can serve as a biocontrol candidate against *B. cereus* in food.

## Highlights

• A novel *B. cereus* phage WAH2 (*Bastillevirus* genus) was isolated and characterized.
• WAH2 shows strong stability under diverse temperature, pH, and NaCl concentration.
• WAH2 inhibits *B. cereus* growth and prevents/disrupts biofilm.
• WAH2 inhibits the outgrowth of germinated spores.
• WAH2 controls *B. cereus* in milk, rice, and pork at both 25 and 4 °C.

## Graphical abstract

**Figure:**
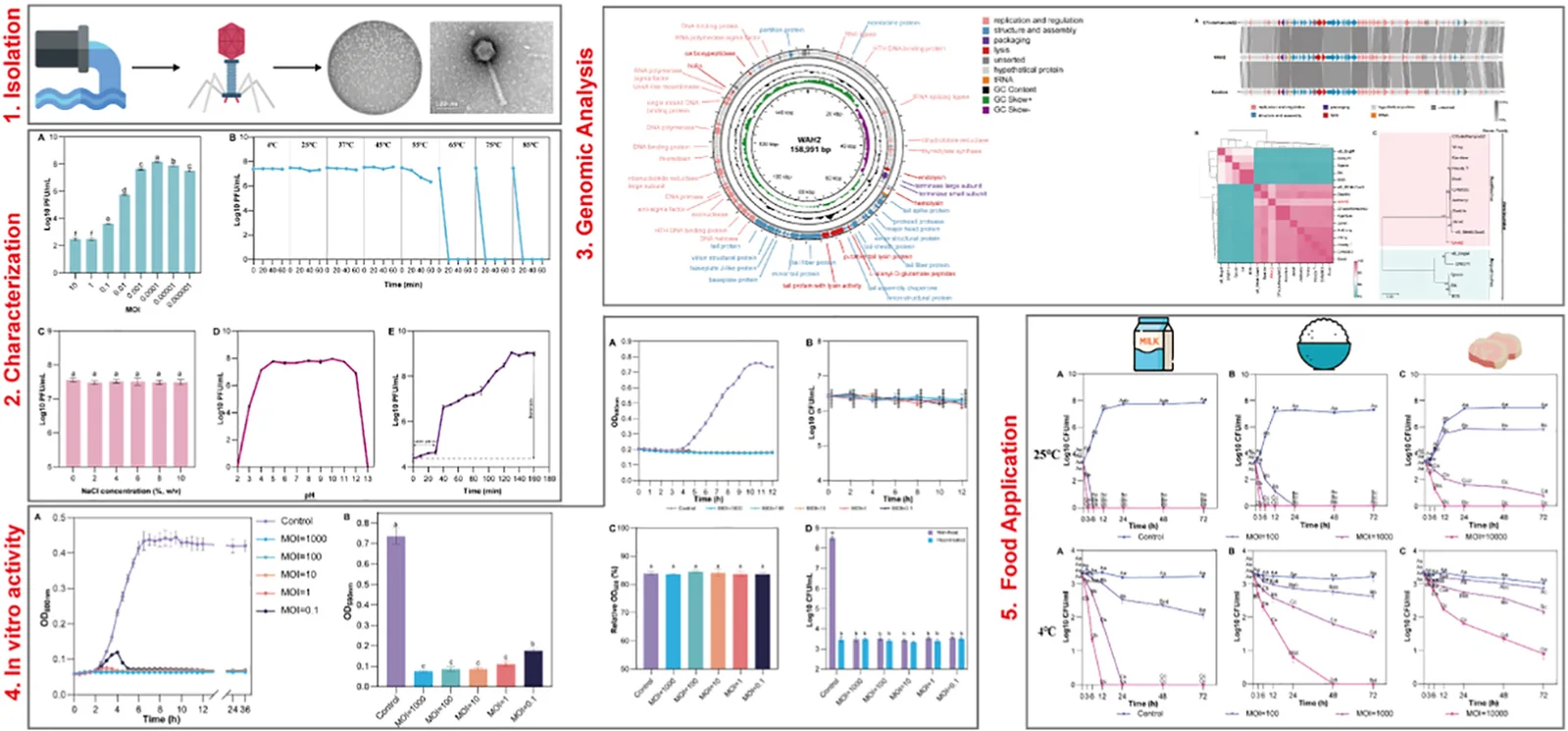

## 1 Introduction

*Bacillus cereus* (*B. cereus*) is a common foodborne pathogen widely found in milk, rice, meat products, and ready-to-eat foods (Enosi Tuipulotu et al., 2021). This bacterium produces enterotoxins and emetic toxins that cause vomiting and diarrhea, posing a serious threat to food safety and public health (Huang et al., 2020). Outbreaks caused by *B. cereus* are frequently reported. Globally, *B. cereus* accounts for approximately 1.4%–12.0% of bacterial foodborne disease outbreaks (Stenfors Arnesen et al., 2008). In Europe, it has been associated with 306 outbreaks and 3192 cases (EFSA & ECDC, 2023); and in China, 419 foodborne outbreaks were reported from 2010 to 2020, resulting in 7892 cases, 2786 hospitalizations, and 5 deaths (Duan et al., 2023). These data highlight that *B. cereus* is a significant food safety risk that should not be ignored.

The ability of *B. cereus* to form spores and biofilms significantly contributes to its environmental adaptability and persistence in food (Huang et al., 2020). Spores exhibit marked resistance to heat, dry, and acid or alkali stress, making them extremely difficult to remove completely from food (Huang et al., 2020). Under favorable conditions, spores germinate into vegetative cells, becoming an important source of subsequent contamination. Furthermore, *B. cereus* readily forms biofilms on food-processing surfaces, further increasing its resistance to disinfectants and other environmental stresses and leading to persistent contamination (Kim et al., 2025a). The rising prevalence of multidrug-resistant strains in food has further increased the difficulty of control (Deng et al., 2021; Hsu et al., 2021; Xiao et al., 2023; Zheng et al., 2024). Although conventional physical and chemical methods can partially mitigate the risk, they are often expensive and may degrade the sensory properties of food (Deng et al., 2020; Ge et al., 2022). Therefore, the development of economical, safe, and effective alternative strategies is urgently needed.

Phages are viruses that specifically infect bacteria. In recent years, they have been regarded as promising biocontrol agents in food due to their low production cost, high host specificity, self-replication ability, and high safety profile (Duc et al., 2020; Ge et al., 2022; Yao et al., 2025). Phages have been applied to control important foodborne pathogens such as *Escherichia coli* (Yamik et al., 2026), *Salmonella* (He et al., 2024; Wong et al., 2020), *Staphylococcus aureus* (Yao et al., 2025), *Listeria monocytogenes* (Byun et al., 2024), *Campylobacter* (Rafiq et al., 2025), and *Clostridium perfringens* (Zhou et al., 2026), demonstrating good efficacy in dairy, meat, and fresh products. However, phages targeting *B. cereus* remain relatively scarce (Abdelaziz et al., 2024; Tan et al., 2023), particularly those with strong lytic ability, robust environmental stability, and high performance in food matrices. Recently, several *B. cereus* phages with biocontrol potential have been reported, including vB_BceS_LY1 (Tan et al., 2023), vB_BceP_LY3 (Tan et al., 2024), DC1/DC2 (Chen et al., 2024), DZ1 (Huang et al., 2024), and Z3 (Li et al., 2024). Although these phages displayed varying lytic activities in food matrices, most exhibited limited efficacy in complex foods and were compromised by the emergence of phage-resistant mutants that led to bacterial regrowth.

In this study, a novel lytic phage, named WAH2, was isolated against *B. cereus* and subjected to comprehensive morphological, biological, and genomic characterization. We further assessed its lytic efficiency, anti-biofilm activity, spore inhibition effect, and biocontrol efficacy in milk, rice, and meat matrices. This study aimed to enrich the phage resource targeting *B. cereus* and provide a scientific basis for phage-based biocontrol strategies in food.

## 2 Materials and methods

### 2.1 Bacterial strains and growth conditions

*B. cereus* strains used in this study were obtained from Chinese sausages isolates reported previously (Xiao et al., 2023). The reference strains ATCC 10876 and CMCC 63303 were acquired from the American Type Culture Collection (ATCC) and National Center for Medical Culture Collection (CMCC), respectively. *B. cereus* BY118LC was used as the host strain for the isolation and propagation of phage WAH2. For host-range determination, *Staphylococcus aureus*, *Staphylococcus borealis*, *Enterococcus faecalis*, *Enterococcus faecium*, *Clostridium perfringens*, *Escherichia coli* and *Salmonella typhimurium* were used as non-target strains to assess phage specificity. All strains were grown in Tryptic Soy Broth (TSB) at 37 °C with shaking at 180 rpm, except *Clostridium perfringens*, which was grown in Reinforced Clostridial Medium (RCM) at 37 °C under anaerobic conditions. Strain information is summarized in Table 1 and Supplementary Table S1.

**Table 1:** Information of *Bacillus cereus* strains and the host range of phage WAH2.

No.	Strain Name	ST^a^	Spot assay^b^	EOP^c^
1	BY3_1LC	745	-	NT
2	BY6_4LC	220	-	NT
3	BY8_3LC	100	-	NT
4	BY9_4LC	/	++	0.468 ± 0.104
5	BY10_3LC	1481	-	NT
6	BY11_3LC	220	-	NT
7	BY12_1LC	24	-	NT
8	BY12_2LC	/	++	0.564 ± 0.085
9	BY22LC	1241	-	NT
10	BY29LC	2833	-	NT
11	BY106LC	/	-	NT
12	BY110LC	4	-	NT
13	BY114LC	745	-	NT
14	BY118LC	2818	++	1.000 ± 0.000
15	BY124LC	/	-	NT
16	BY132LC	2821	+	0.013 ± 0.003
17	BY133LC	2832	-	NT
18	TH38LC	/	-	NT
19	TH39_1LC	/	-	NT
20	TH69LC	745	-	NT
21	TH145_1LC	2839	-	NT
22	TH145_2LC	1481	-	NT
23	TH148LC	2840	-	NT
24	TH156LC	2840	-	NT
25	TH163_2LC	/	-	NT
26	TH164LC	/	-	NT
27	TH167_1LC	142	-	NT
28	TH173LC	2641	-	NT
29	TH202_2LC	1465	-	NT
30	TH203_2LC	/	+	0.019 ± 0.003
31	TH204_2LC	24	-	NT
32	TH223LC	2741	-	NT
33	TH226LC	1601	-	NT
34	TH229_2LC	4	-	NT
35	TH229_3LC	1601	-	NT
36	TH230_2LC	395	-	NT
37	TH231_1LC	2150	-	NT
38	TH232LC	395	+	0.096 ± 0.018
39	TH235LC	24	-	NT
40	TH240LC	24	-	NT
41	TH243_2LC	2150	-	NT
42	TH245LC	/	-	NT
43	TH248_3LC	24	-	NT
44	TH256LC	2641	-	NT
45	CB85LC	4	-	NT
46	CS50LC	395	-	NT
47	LD93LC	24	-	NT
48	LD112LC	/	-	NT
49	WG12HT	/	-	NT
50	ATCC 10876	/	++	0.237 ± 0.035
51	CMCC 63303	/	-	NT

a “ST”, the abbreviation of sequence type; “/”, ST not available.

b “++”, the clear lysis zone; “+”, the turbid lysis zone; and “-”, no lysis.

c “EOP”, the abbreviation of efficiency of plating; “NT”, not tested; Values are presented as mean ± SD (n = 3).

### 2.2 Phage isolation and purification

Phage WAH2 was isolated from a sewage sample collected at a food factory wastewater treatment facility in Guangzhou, China. Phage isolation was carried out with slight modifications to a previously reported method (Van Twest and Kropinski, 2009). Briefly, the sample was first centrifuged at 5000×g for 10 min to remove particulate matter. The supernatant was then passed through a series of membrane filters (5.0, 1.2, 0.45, and 0.22 μm) to remove residual bacteria. Next, the filtered sample was concentrated in a 100 kDa ultrafiltration tube (Millipore, USA) by centrifugation at 5000×g for 20 min. For phage enrichment, 100 μL of the concentrated sample was mixed with 100 μL of host strain BY118LC in 2 mL of TSB and incubated overnight at 37 °C with shaking at 180 rpm. After incubation, the culture was centrifuged at 12000×g for 10 min, and the supernatant was filtered through a 0.22 μm membrane. The resulted lysate was then subjected to plaque purification by the double-layer agar method. After overnight incubation at 37 °C, a single plaque was picked and suspended in SM buffer (100 mM NaCl, 8 mM MgSO_4_, 50 mM Tris–HCl pH 7.5). The purification step was repeated three times to obtain a clonal phage stock, which was stored at 4 °C for later use.

### 2.3 Transmission electron microscopy (TEM)

To examine the morphology of phage WAH2, a phage suspension with a titer of approximately 10^8^ PFU/mL was prepared for TEM analysis. A small volume of the suspension was placed on copper grids and kept at room temperature for 15 min to allow adsorption. Excess liquid was then removed, and the grids were negatively stained with 2.0% (w/v) phosphotungstic acid (pH 7.0) for 10 min. After air-drying, the samples were observed using a TEM (Thermo Fisher, Hillsboro, USA) at an accelerating voltage of 80 kV.

### 2.4 Phage host range and efficiency of plating (EOP) assay

The host range of phage WAH2 was initially screened against 51 *B. cereus* strains using a spot assay. In addition, WAH2 was tested against representative non-target bacteria (Supplementary Table S1). For each strain, 100 μL of bacterial culture was mixed with 5 mL semi-solid agar and poured onto a solid agar plate. After solidification, 5 μL of phage suspension (∼10^8^ PFU/mL) was spotted onto the surface of each lawn. The plates were then incubated overnight at 37 °C. For *C. perfringens*, spot assays were performed under anaerobic conditions. Phage sensitivity was assessed according to the appearance of the lytic zones, which were classified as clear lysis zones (++), turbid lysis zones (+), or no lysis (−). Subsequently, the efficiency of plating (EOP) for all spot-assay-positive strains was determined using double-layer agar method. EOP was calculated as the ratio of PFU/mL on the test strain to that on the propagation host strain BY118LC. All assays were performed in three independent replicates. The strains tested and their EOP values are listed in Table 1.

### 2.5 Multiplicity of infection (MOI) assay

To determine the optimal MOI for phage WAH2, exponential-phase host cells and phage were co-incubated at MOIs ranging from 10 to 0.000001. After 4 h of incubation at 37 °C, the cultures were centrifuged at 12000×g for 5 min to remove cells. The phage yield in the resulting supernatant was then measured by the double-layer agar method. The MOI that produced the highest phage titer was selected as the optimal MOI. Each assay was performed in triplicate.

### 2.6 Stability tests of phage

The stability of phage WAH2 was evaluated under different environmental conditions. To test thermal stability, 100 μL of phage suspension (∼10^7^ PFU/mL) was added to 900 μL of SM buffer and exposed to 4, 25, 37, 45, 55, 65, 75, and 85 °C. Aliquots were taken at 0, 20, 40, and 60 min during incubation. For pH stability, the phage was incubated in SM buffer adjusted to pH 2–13 and incubated at 37 °C for 1 h. To assess salt tolerance, the phage was incubated in SM buffer containing NaCl at final concentrations of 2, 4, 6, 8, or 10% (w/v) at 37 °C for 1 h. The remaining phage titer after each treatment was measured by the double-layer agar method. Each experiment was carried out in triplicate.

### 2.7 One-step growth curve and phage adsorption

The lytic cycle of phage WAH2 was examined by a one-step growth experiment. Phage and host cells were first combined at an MOI of 0.1 and kept stationary at 37 °C for 10 min to allow adsorption. The mixture was then centrifuged at 8000×g for 5 min, and the pellet was resuspended in 10 mL of fresh TSB. After that, the culture was incubated at 37 °C with shaking at 180 rpm. At 10 min intervals, 300 μL samples were removed, passed through a 0.22 μm membrane, and used for phage titration by the double-layer agar method. Burst size (P_b_) was calculated using the following equation: P_b_ = P/B_0_, where P is the phage titer at the plateau phase and B_0_ is the initial number of infected bacteria. Phage adsorption was assessed by incubating 5 mL of host bacterial culture with 100 μL of phage suspension at an MOI of 0.01. At 3, 5, 10, 15, and 20 min, aliquots were collected and centrifuged (8000 × g, 1 min). The supernatants were filtered through 0.22 μm membranes, and the filtrates were used to quantify residual unadsorbed phages. A bacteria-free control was used to determine the initial phage titer (P_0_). The phage adsorption rate was calculated as: adsorption rate (%) = (P_0_**−**P_t_)/P_0_ × 100, in which P_t_ is the residual unadsorbed phage titer at time t. All assays were performed in triplicate.

### 2.8 Whole-genome sequencing and analysis

Phage particles were recovered from the lysate by overnight precipitation at 4 °C with 10% (w/v) polyethylene glycol 8000 and 1 M NaCl. The precipitated material was collected by centrifugation at 10000×g for 15 min at 4 °C and then resuspended in SM buffer. To eliminate contaminating bacterial nucleic acids, the concentrated phage preparation was treated with DNase I and RNase A, each at 100 U/mL (Sangon Biotech, Shanghai, China). Phage genomic DNA was subsequently extracted using the Omega viral DNA kit (Omega Bio-Tek, USA) according to the manufacturer's protocol.

Genome sequencing was carried out on the Illumina Novaseq 6000 platform (Illumina, San Diego, USA). After quality filtering, the reads were assembled de novo using SPAdes v. 4.2.0 (Bankevich et al., 2012). Open reading frames (ORFs) were predicted and annotated using Bakta (Schwengers et al., 2021). Putative protein-coding sequences were checked by BLASTp against the non-redundant protein database, and the best-scoring matches were used for functional assignment. The presence of tRNA genes, antibiotic resistance genes, and virulence factors were screened with tRNAscan-SE (Chan et al., 2021), the Comprehensive Antibiotic Resistance Database (CARD) (Alcock et al., 2023), and the Virulence Factor Database (VFDB) (Liu et al., 2022), respectively. A genome map was generated using Proksee (Grant et al., 2023).

For comparative genomic analyses, the WAH2 genome was queried against the NCBI nucleotide database using BLASTn. Similarities with related phages were visualized with Easyfig (Sullivan et al., 2011). Pairwise intergenomic similarities were calculated with the Virus Intergenomic Distance Calculator (VIRIDIC) (Moraru et al., 2020), and the resulting similarity matrix was used to construct a heatmap illustrating the phylogenetic relationships among phages via R software. Phylogenetic analysis of the large terminase subunit was performed after amino acid sequence alignment with Clustal W. A neighbor-joining tree was then constructed in MEGA 11 with 1000 bootstrap replicates (Tamura et al., 2021).

### 2.9 Growth inhibition experiment

Growth inhibition by phage WAH2 was assessed by recording bacterial growth in a 96-well plate. Host cells were exposed to phage at different MOIs (1000, 100, 10, 1, and 0.1), while a phage-free bacterial suspension was used as the control. The plate was incubated at 37 °C, and OD_600_ values were measured with a microplate reader (PE Ensight, Revvity, USA). Measurements were taken every 30 min for the first 12 h, followed by additional readings at 24 and 36 h. All experiments were performed in triplicate.

### 2.10 Biofilm prevention and disruption assays

Biofilm prevention by phage WAH2 was evaluated by crystal violet (CV) assay with minor modifications to a previously reported method (Chen et al., 2024). In a 96-well plate, host bacteria (∼10^6^ CFU/mL) were exposed to phage at different MOIs (1000, 100, 10, 1, 0.1) and incubated at 37 °C for 24 h. A phage-free bacterial suspension was used as the control. After incubation, each well was washed three times with PBS to remove planktonic cells. The attached biofilm was then stained with 0.1% (w/v) CV for 15 min. The excess dye was removed by washing three times with ddH_2_O, and the plate was air-dried. Finally, the bound dye was dissolved in 33% (v/v) acetic acid, and the OD_590_ was measured with a microplate reader (PE Ensight, Revvity, USA).

The effect of phage WAH2 on biofilm disruption on stainless steel surfaces was assessed using a previously reported method (Kang et al., 2026) with minor modifications. Type 304 stainless steel coupons (16 mm diameter, 1.8 mm thick) were cleaned with 75% ethanol, sterilized by autoclaving, and placed in the wells of a 12-well plate containing 2 mL of TSB per well. Each well was inoculated with 20 μL of host bacteria (∼10^8^ CFU/mL) and incubated at 37 °C for 48 h to allow mature biofilm formation. After the coupons were washed with PBS to remove planktonic cells, they were transferred to fresh wells containing 2 mL of TSB supplemented with WAH2 at 10^7^ or 10^8^ PFU/mL and incubated at 37 °C for 12 h. TSB without phage served as the control. Biofilm biomass on the coupons was quantified by CV staining and OD_590_ measurement as described above. For viable cell counts, coupons were vortexed for 2 min and sonicated for 5 min (45 kHz) in PBS with sterile glass beads. The resulting suspension was serially diluted and plated onto TSA. All experiments were performed in triplicate.

### 2.11 Spore suspension preparation

Spore preparation was performed with minor modifications to a previously described protocol (Zhang et al., 2024). Briefly, *B. cereus* was cultured overnight at 37 °C with shaking at 180 rpm, and 100 μL of the culture was spread onto manganese nutrient agar plates (Hopebio, China) and incubated at 37 °C for 5 days. After confirming the sporulation rate exceeded 95% by spore staining, spores were harvested, washed five times by centrifugation at 6000 rpm for 10 min and resuspended in sterile water. To eliminate residual vegetative cells, the suspension was heat-treated at 80 °C for 10 min, followed by three additional washes. The purified spore suspension was stored at 4 °C for subsequent experiments.

### 2.12 Spore inhibition assay

The inhibitory effect of phage on *B. cereus* spores was evaluated using a microplate reader (PE Ensight, Revvity, USA). Phage was added to TSB containing 10^6^ CFU/mL of spores at various MOIs (1000, 100, 10, 1, 0.1). Then, 200 μL of each mixture was transferred into individual wells of a 96-well plate, with wells containing spores in TSB without phage served as controls. The plate was incubated at 37 °C, and the OD_600_ was monitored every 30 min for 12 h. Additionally, after 12 h of incubation, samples from each group were collected and divided into two aliquots. One was plated directly after serial dilution, and the other was heat-shocked at 80 °C for 10 min before dilution and plating. All experiments were performed in triplicate.

### 2.13 Time-kill curve

To evaluate the bactericidal kinetics of phage against *B. cereus* spores, spores (10^6^ CFU/mL) in a 0.85% NaCl solution were treated with phage at various MOIs (1000, 100, 10, 1, 0.1). Spore without phage served as the control. The mixtures were incubated at 37 °C, and samples were collected at 0, 2, 4, 6, 8, and 12 h. The number of surviving spores was determined using serial dilution and plating.

### 2.14 Spore germination assay

The effect of phage on *B. cereus* spore germination was evaluated by monitoring the changes in OD_600_ (Zhang et al., 2024). Prior to the assay, the spores were heat-activated at 70 °C for 15 min. The activated spore (10^6^ CFU/mL) in TSB were then mixed with the phage at various MOIs (1000, 100, 10, 1, 0.1). Subsequently, 200 μL of each mixture was transferred into a 96-well plate, with phage-free mixtures as the control. The plate was incubated at 37 °C, and OD_600_ was measured at 0 h (OD_0h_) and 1 h (OD_1h_). The relative OD_600_ was calculated using the following equation: Relative OD_600_ (%) = (OD_1h_/OD_0h_)×100%. All experiments were performed in triplicate.

### 2.15 Application of phage in food matrices

To assess antibacterial efficacy of phage WAH2 in food matrices, UHT whole milk, rice, and pork were used as model matrices at 4 and 25 °C. All samples were purchased from a local supermarket in Guangzhou, China. For rice, the grains were milled into powder, and 1 g of powder was suspended in 50 mL of sterile water to prepare a 2% (w/v) suspension, which was then autoclaved. Pork was cut into uniform pieces (1 g/piece), surface-sterilized with 75% ethanol, air-dried, and exposed to UV light for 30 min.

For the milk and rice assays, 4.8 mL of each sterile matrix was mixed with 100 μL of *B. cereus* BY118LC and 100 μL of phage suspension. The final bacterial concentration was approximately 10^3^ CFU/mL. Phage doses corresponded to MOIs of 100, 1000, and 10000, corresponding to final phage concentrations of approximately 10^5^, 10^6^, and 10^7^ PFU/mL, respectively. MOI was calculated as the ratio of total PFU to total CFU after mixing with the food matrix. For pork, 100 μL of bacterial suspension was applied to the surface and allowed to attach for 30 min at room temperature before phage treatment, resulting in a final concentration of 10^3^ CFU/g. Then, 100 μL of phage suspension was added at the same MOIs as above, corresponding to approximately 10^5^, 10^6^, and 10^7^ PFU/g, respectively. Samples containing bacteria but no phage were used as controls. All groups were incubated at 4 or 25 °C, and bacterial counts were enumerated at 0, 3, 6, 12, 24, 48, and 72 h by serial dilution. Pork-associated bacteria were recovered by vortexing the samples in PBS-filled centrifuge tubes. Each experiment was performed in triplicate.

### 2.16 Phage persistence and resistance in TSB and UHT whole milk

The persistence of phage WAH2 and the emergence of phage-resistant survivors were assessed in TSB and UHT whole milk at 25 °C and 4 °C. For persistence, phage lysate was diluted in TSB or UHT milk to approximately 10^7^ PFU/mL, dispensed into sterile tubes, and stored statically at the respective temperatures. At 0, 24, 48, 72, 96, and 120 h, samples were collected, centrifuged, filtered, and infectious phage titers were determined by the double-layer agar method. For resistance, *B. cereus* BY118LC was grown overnight in TSB at 37 °C, harvested, washed twice with PBS, and resuspended in TSB or UHT milk to approximately 10^4^ CFU/mL. Each matrix was divided into two groups: a phage-treated group with WAH2 at an MOI of 100, and a control group with an equal volume of SM buffer. The mixtures were incubated statically at 25 °C or 4 °C. At 0, 24, 48, 72, 96, and 120 h, samples were serially diluted in PBS and plated onto TSA without phage (total viable cells) and TSA containing WAH2 at a final concentration of 10^8^ PFU/mL (resistant survivors). Plates were incubated at 37 °C and colonies were counted. The resistance frequency was calculated as CFU/mL on phage-containing plates divided by CFU/mL on phage-free plates; when no colonies were detected, the frequency was recorded as below the limit of detection. All experiments were performed in triplicate.

### 2.17 Statistical analysis

GraphPad Prism 8 (GraphPad Software, San Diego, California, USA) was used for all statistical analyses. Data are presented as the mean ± SD. When two groups were compared, an unpaired *t*-test was applied. For the food matrix experiments, two-way repeated-measures ANOVA followed by Tukey's post hoc test was performed. A *P* ≤ 0.05 was considered statistically significant. Different letters indicate significant differences among treatments.

### 2.18 Data availability

The complete nucleotide sequence of *Bacillus* phage WAH2 has been deposited in the GenBank database under Accession Number PZ137004.

## 3 Results

### 3.1 Isolation and morphological characterization of phage WAH2

Phage WAH2 was isolated using *B. cereus* strain BY118LC as the host. This strain, which had been isolated from Chinese sausages, showed resistance to multiple antibiotics, including penicillin, amoxicillin, cefoxitin, tiamulin, ceftiofur, sulfamethoxazole-trimethoprim, and tigecycline. It also carried several virulence factors, including *entFM*, *nheABC*, *hblABCD*, *cytK*, and *bceT* (Xiao et al., 2023). Given these features, BY118LC was selected as the indicator host for phage isolation. On the bacterial lawn, WAH2 produced clear, uniform, and circular plaques with an average diameter of 1.78 ± 0.18 mm (Fig. 1A). TEM analysis showed an icosahedral capsid measuring 88.28 ± 2.11 nm in diameter and a contractile tail of 199.44 ± 6.62 nm in length (Fig. 1B). These morphological traits are consistent with a *myovirus*-like morphology, and the taxonomic classification of WAH2 was further determined by whole-genome sequencing and phylogenetic analysis.

**Fig. 1:**
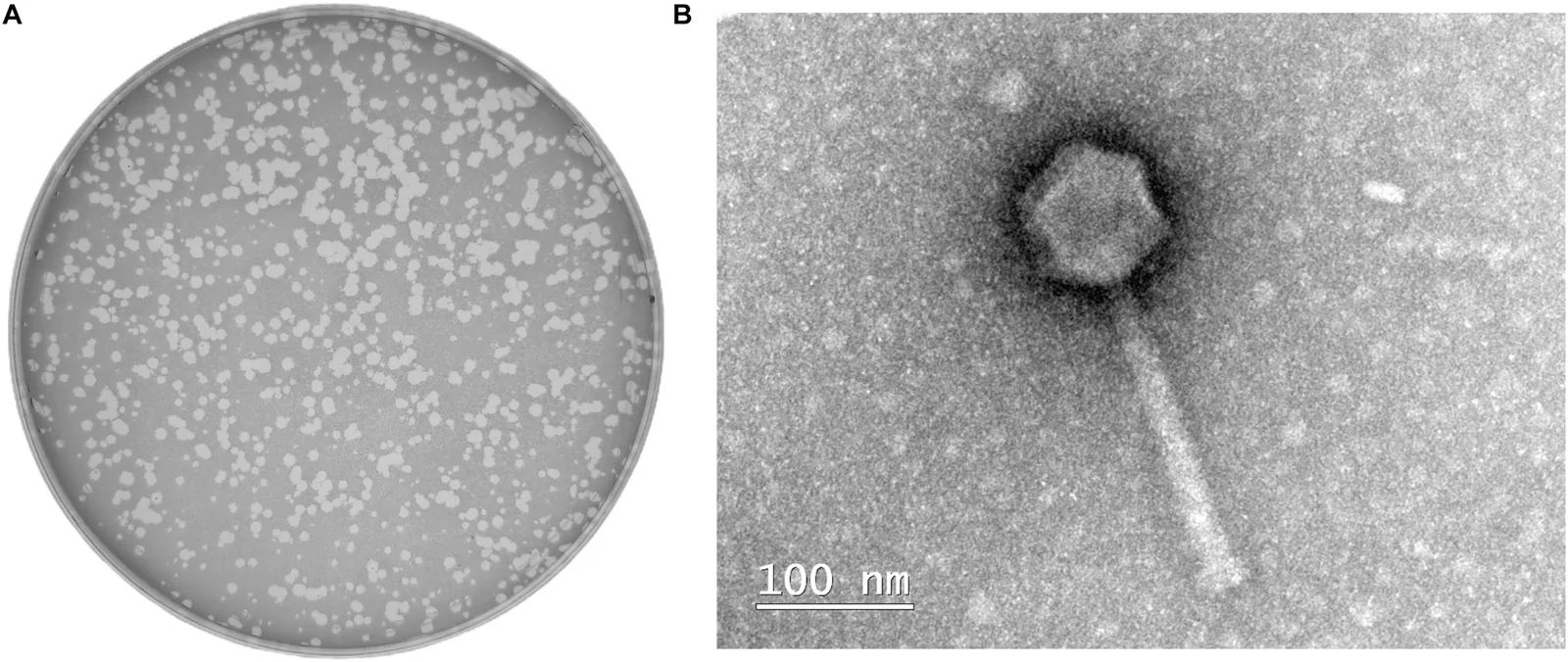
Morphological features of phage WAH2. (A) Plaque formation on a double-layer agar plate. (B) Transmission electron micrograph of WAH2 (scale bar = 100 nm).

### 3.2 Host range and efficiency of plating of phage WAH2

The host range of WAH2 was screened against 51 *B. cereus* strains. Spot assay showed lysis zones for 7 strains (13.7%) (Table 1). Of these, 4 strains showed clear lysis zones and 3 showed turbid lysis zones. All seven spot-positive strains produced visible plaques in the double-layer agar assay, confirming productive infection. EOP values relative to the propagation host BY118LC ranged from 0.013 to 0.564 (Table 1). The susceptible strains were distributed among 3 distinct sequence types (STs), namely ST2818, ST2821, and ST395. WAH2 did not form lysis zones on any of the tested non-target bacteria (Supplementary Table S1), supporting its narrow lytic spectrum.

### 3.3 Biological characteristics of phage WAH2

The optimal MOI for WAH2 was identified as 0.0001, yielding the highest titer of 1.6 × 10^8^ PFU/mL (Fig. 2A). Thermal stability assays indicated that WAH2 remained active from 4 to 45 °C, with no significant loss in titer. However, exposure to 55 °C triggered a time-dependent decline in titer, with reductions of approximately 0.2, 0.8, and 1.2 log_10_ PFU/mL after 20, 40, and 60 min, respectively. Complete inactivation was observed at 65 °C and above (Fig. 2B). WAH2 also showed stable activity across NaCl concentrations ranging from 2% to 10% (Fig. 2C). In addition, it maintained high viability from pH 5 to 11, but was fully inactivated at pH 2 and 13. Moderate titer reductions (3.2, 0.5, and 0.8 log_10_ PFU/mL) were observed at pH 3, 4, and 12, respectively (Fig. 2D). A one-step growth assay showed that WAH2 had a latent period of 30 min and a burst size of 208 PFU/cell (Fig. 2E). WAH2 adsorbed rapidly, with approximately 44.05% adsorption within 5 min, reaching 78.89% at 20 min (Fig. 2F).

**Fig. 2:**
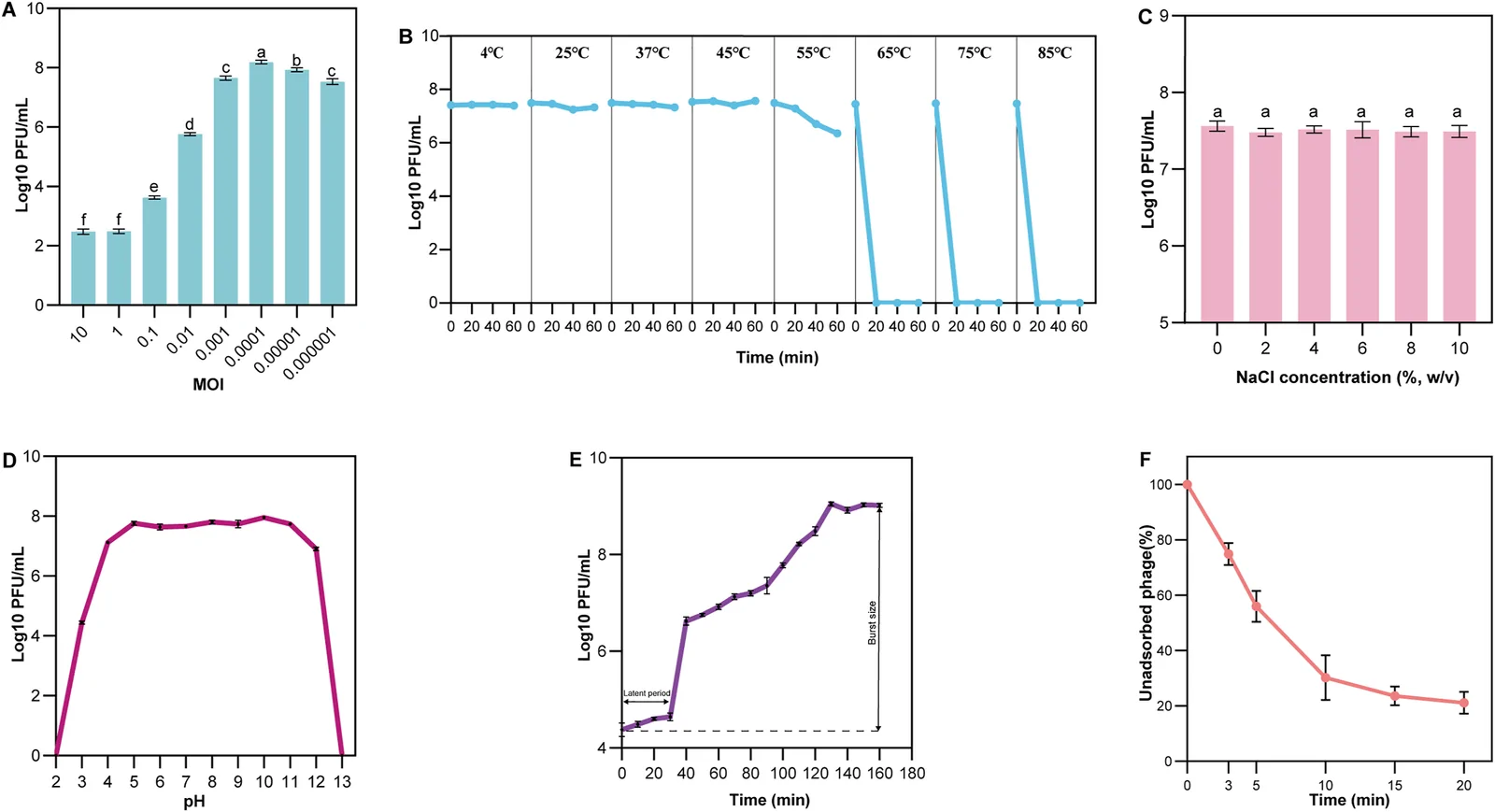
Biological characterization of phage WAH2. (A) Multiplicity of infection (MOI). (B) Thermal stability. (C) NaCl stability. (D) pH stability. (E) One-step growth curve. (F) Adsorption rates. Data are presented as the mean ± SD. Distinct letters indicate statistically significant differences (*P* < 0.05).

### 3.4 Genome and phylogenetic analysis of phage WAH2

The WAH2 genome is a 158991 bp linear double-stranded DNA molecule with a GC content of 37.96% (Fig. 3). Genomic annotation predicted 284 ORFs, including 71 with assigned functions and 213 hypothetical proteins. The functional ORFs were grouped into modules related to DNA replication and regulation, structure and assembly, packaging, and lysis (Supplementary Table S2). The genome also contains 6 tRNA genes. Notably, the absence of antibiotic resistance genes, virulence factor, or lysogeny-related elements underscores the biosafety of WAH2 for food applications.

**Fig. 3:**
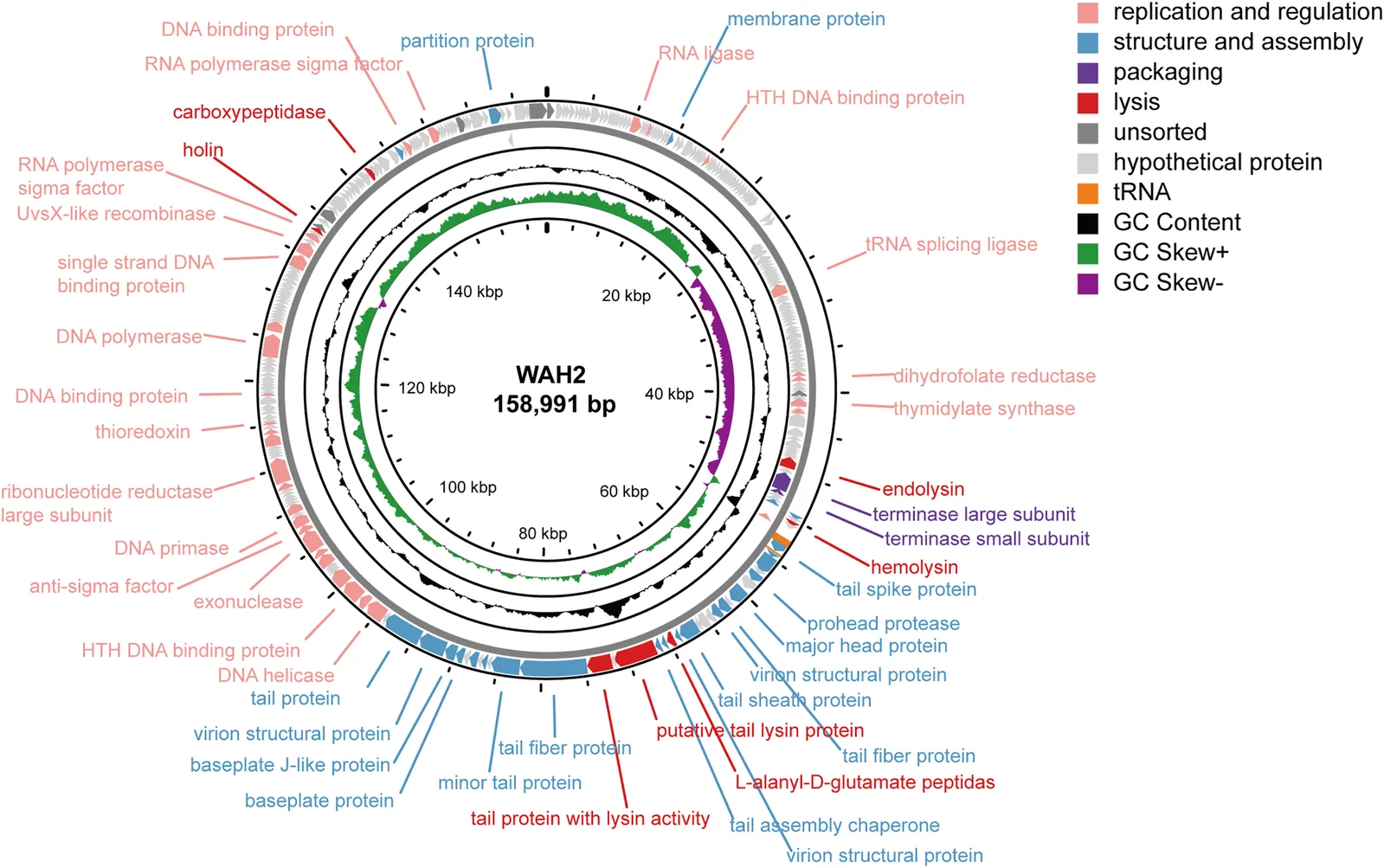
Circular genomic organization of phage WAH2. The map displays the annotated genome layout. The circles, ordered from the center outward, indicate GC skew, GC content, and ORFs transcribed in both orientations. Functional categories are color-coded as follows: replication and regulation (pink); structure and assembly (blue); packaging (purple); lysis (red); unsorted (dark gray); hypothetical proteins (light gray).

BLASTn analysis showed that WAH2 shared 92.47% nucleotide identity (92% coverage) with *Bacillus* phage OTooleKemple52 and Kamfam. Comparison genomic alignment further confirmed that WAH2 maintains a conserved architecture with these phages, with variations primarily localized within the hypothetical protein clusters (Fig. 4A). Intergenomic similarity analysis using VIRIDIC was performed on 15 related phages with query coverage >19% (Supplementary Table S3). The resulting heatmap segregated the phages into two distinct clusters, and WAH2 grouped closely with OTooleKemple52, Kamfam, and Bastille (Fig. 4B). A phylogenetic tree based on the large terminase subunit placed WAH2 within the genus *Bastillevirus*(Fig. 4C). Taken together, these results support the assignment of WAH2 as a novel species within the genus *Bastillevirus*, family *Herelleviridae*.

**Fig. 4:**
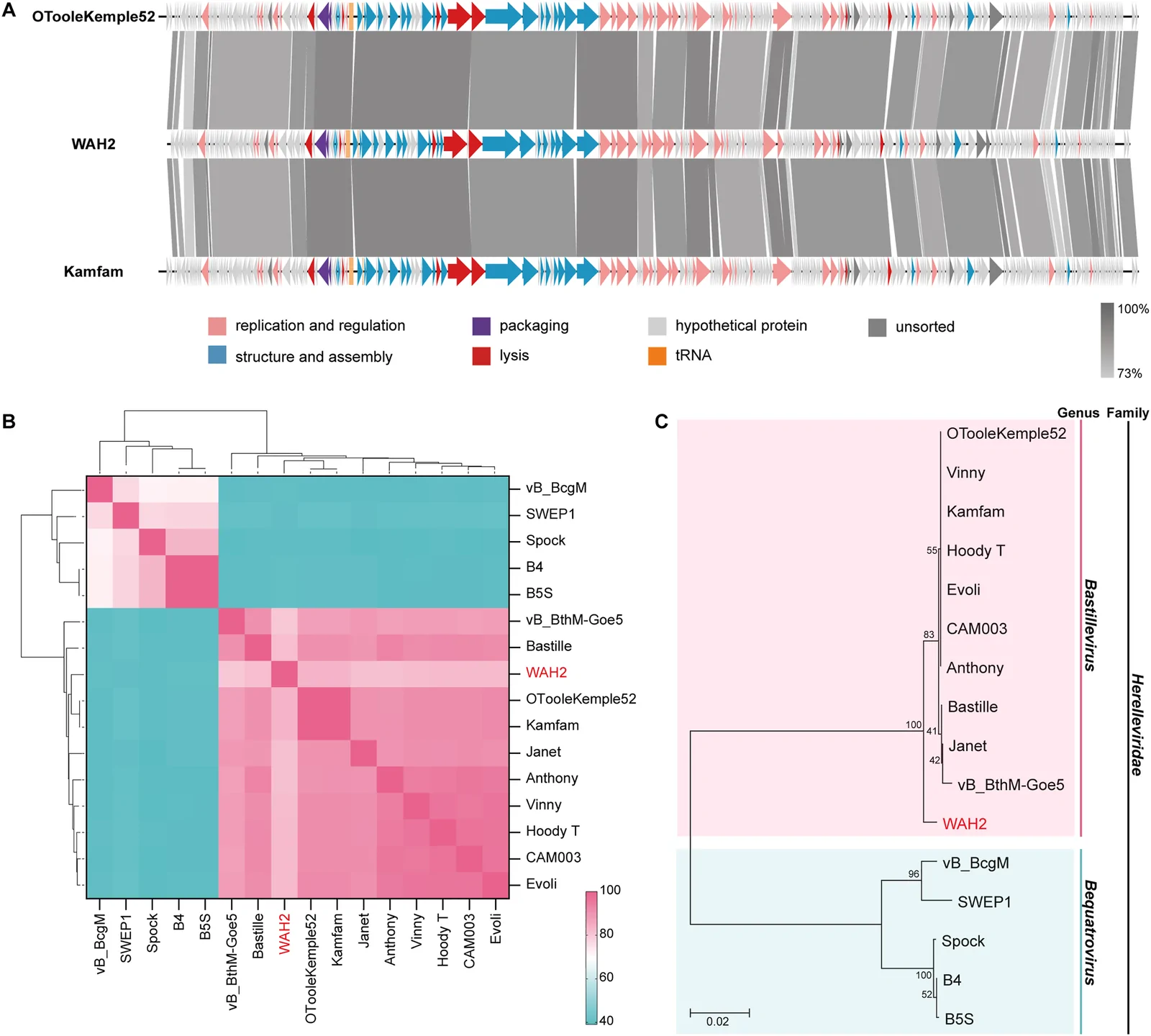
Comparative genomic and phylogenetic analysis of phage WAH2. (A) Nucleotide homology comparison of the WAH2 genome. ORFs are color-coded by function, and gray-shaded regions represent sequence homology (darker shading indicates higher identity). (B) Heatmap displaying intergenomic nucleotide similarity between WAH2 and related phages. Similarity values were calculated using VIRIDIC. The color scale (blue to red) reflects genomic similarity, with WAH2 highlighted in red. (C) Phylogenetic analysis based on the amino acid sequences of the large terminase subunit. WAH2 is marked in red text. All depicted phages belong to the *Herelleviridae* family, with distinct clustering into the genera *Bastillevirus* and *Bequatrovirus*.

### 3.5 Lytic activity, biofilm prevention, and biofilm disruption of phage WAH2

Phage WAH2 showed a clear dose-dependent inhibitory effect on *B. cereus* BY118LC in TSB. At MOIs of 1000, 100, and 10, bacterial growth was fully suppressed throughout the 36 h period. At MOIs of 1 and 0.1, although minor initial growth was observed, the bacteria subsequently arrested proliferation, with no regrowth detected by the end of the assay (Fig. 5A). Furthermore, WAH2 markedly inhibit biofilm formation. At MOIs of 1000, 100, 10, 1, and 0.1, biofilm mass (OD_590_) was reduced by 0.66 ± 0.04, 0.65 ± 0.03, 0.65 ± 0.04, 0.62 ± 0.05, and 0.57 ± 0.05, respectively (Fig. 5B). In addition, WAH2 effectively disrupted pre-established biofilms on stainless steel coupons. Biofilms were pre-formed for 48 h and then treated with WAH2 at 10^7^ and 10^8^ PFU/mL for 12 h. At these concentrations, biofilm mass (OD_590_) was reduced by 1.07 ± 0.04 and 0.99 ± 0.07, respectively (Fig. 5C). Consistenly, viable biofilm-associated cells were reduced by 3.84 ± 0.06 and 3.68 ± 0.19 log_10_ CFU/mL, respectively (Fig. 5D).

**Fig. 5:**
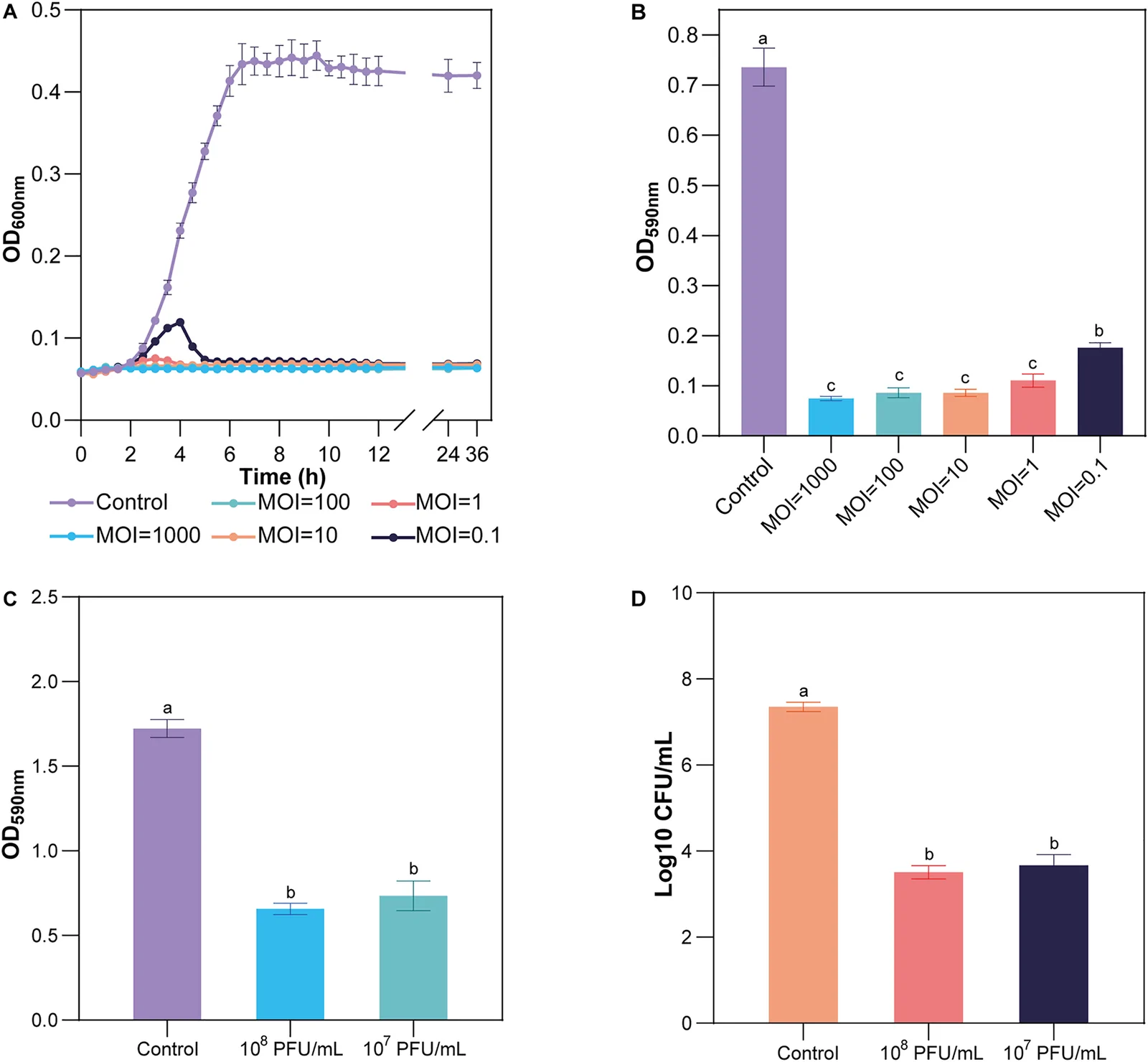
*In vitro* antibacterial and antibiofilm efficacy of phage WAH2. (A) Growth inhibition of *B*. *cereus* BY118LC in TSB at various MOIs (1000, 100, 10, 1, 0.1). (B) Prevention of biofilm formation by BY118LC in 96-well plates after 24 h of incubation at different MOIs (1000, 100, 10, 1, 0.1), biofilm biomass was measured by crystal violet (OD_590_). (C) Disruption of pre-established biofilms on stainless steel coupons by WAH2 at 10^7^ and 10^8^ PFU/mL. Biofilms were pre-formed for 48 h, and then treated with WAH2 for 12 h; biofilm biomass was measured by crystal violet (OD_590_). (D) Viable biofilm-associated cells after WAH2 treatment at 10^7^ and 10^8^ PFU/mL under the same condition as in (C). Data are presented as mean ± SD. Statistically significant differences (*P* < 0.05) are indicated by different letters.

### 3.6 Phage WAH2 inhibits the outgrowth of germinated spores

To evaluate the effect of WAH2 on *B. cereus* spores, the growth curve was monitored in TSB medium. The results showed that spores in the control group germinated and grew normally, reaching stationary phase after about 10 h. In contrast, the phage-treated groups at all tested MOIs showed no increase in OD_600_ within 12 h, indicating that WAH2 effectively inhibited spore growth under nutrient conditions (Fig. 6A). The direct killing effect of WAH2 on dormant spores was then examined in 0.85% NaCl solution. Time-kill curves showed no obvious change in bacterial counts in either the phage-treated or the control groups throughout the observation period, indicating that WAH2 did not inactivate dormant spores (Fig. 6B). In addition, the germination assay showed no significant difference in OD_600_ changes between the phage-treated and the control groups, indicating that WAH2 did not affect spore germination (Fig. 6C). Finally, samples from the 12 h cultures were plated with and without heat treatment. In the control group, the bacterial counts without heat treatment were about 4 Log10 CFU/mL higher than that with heat treatment. In the phage-treated groups, there was no significant difference between the counts with and without heat treatment. These results indicate that WAH2 inhibited the outgrowth of germinated spores (Fig. 6D).

**Fig. 6:**
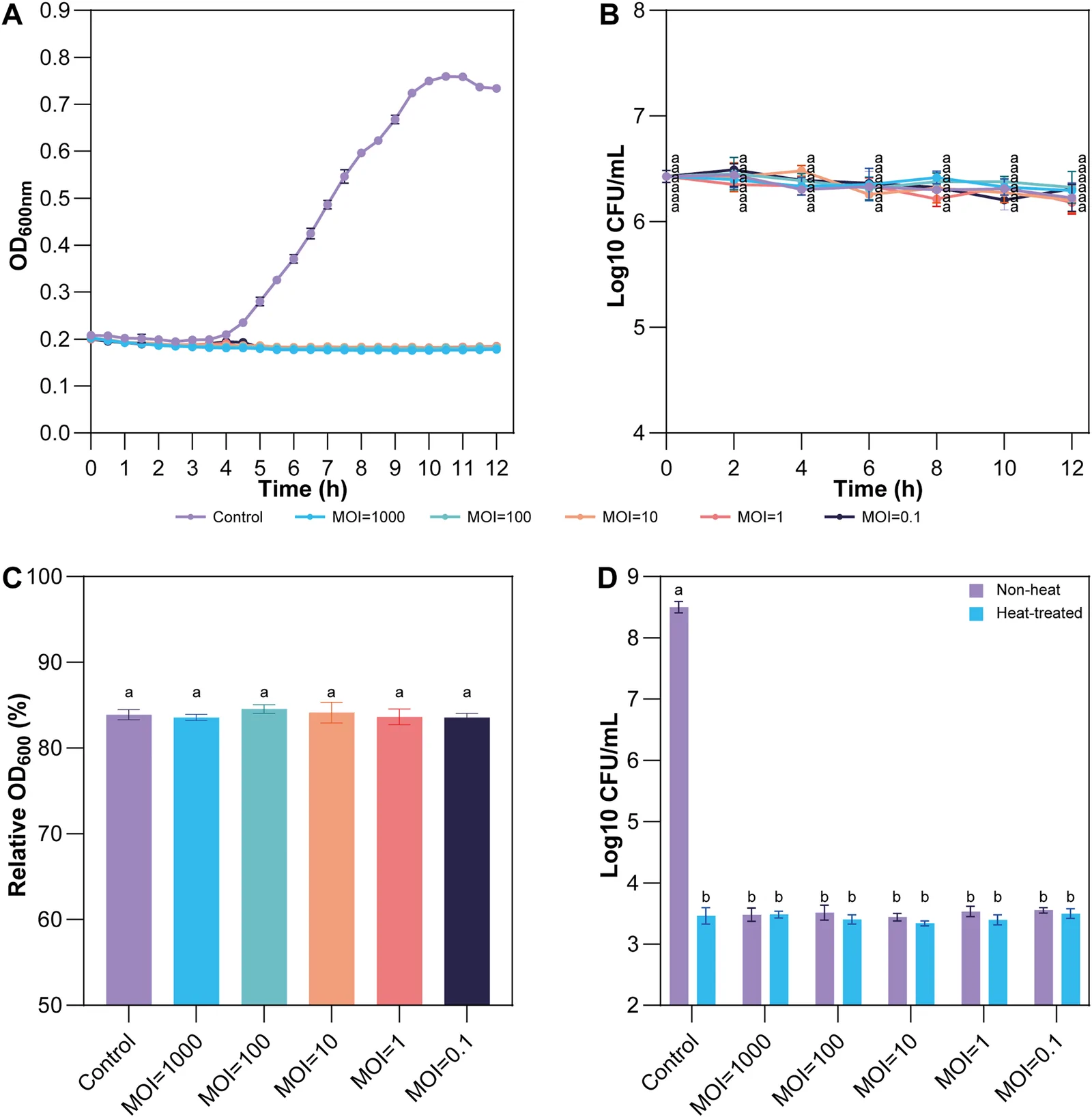
Inhibitory effect of phage WAH2 on *B*. *cereus* spores. (A) Growth curves of *B. cereus* spores treated with WAH2 at different MOIs in TSB medium. (B) Time-kill curves of WAH2 against dormant spores in 0.85% NaCl solution. (C) Effect of WAH2 on spore germination, as indicated by changes in OD_600_. (D) Viable counts of samples collected after12 h incubation with and without heat treatment. Data are shown as mean ± SD. Different letters indicate statistically significant differences (*P* < 0.05).

### 3.7 Biocontrol efficacy of phage WAH2 in diverse food matrices

The antibacterial efficiency of WAH2 was evaluated in milk, rice, and pork at 25 and 4 °C. At 25 °C, bacterial populations in the untreated controls exhibited rapid proliferation, whereas bacterial numbers in the phage-treated groups declined in a dose-dependent manner (Fig. 7). WAH2 showed strong killing activity in all three food matrices. In milk, bacterial counts dropped below the detection limit within 3 h at MOIs of 10000 and 1000, and within 6 h at an MOI of 100 (Fig. 7A). In rice, the same MOIs achieved undetectable levels at 3, 6, and 24 h, respectively (Fig. 7B). In pork, fully elimination was reached by 12 h at an MOI of 10000, while MOIs of 1000 and 100 caused reductions of 6.68 and 1.66 log_10_ CFU/g at 72 h, respectively (Fig. 7C). No bacterial regrowth was observed during 72 h period.

**Fig. 7:**
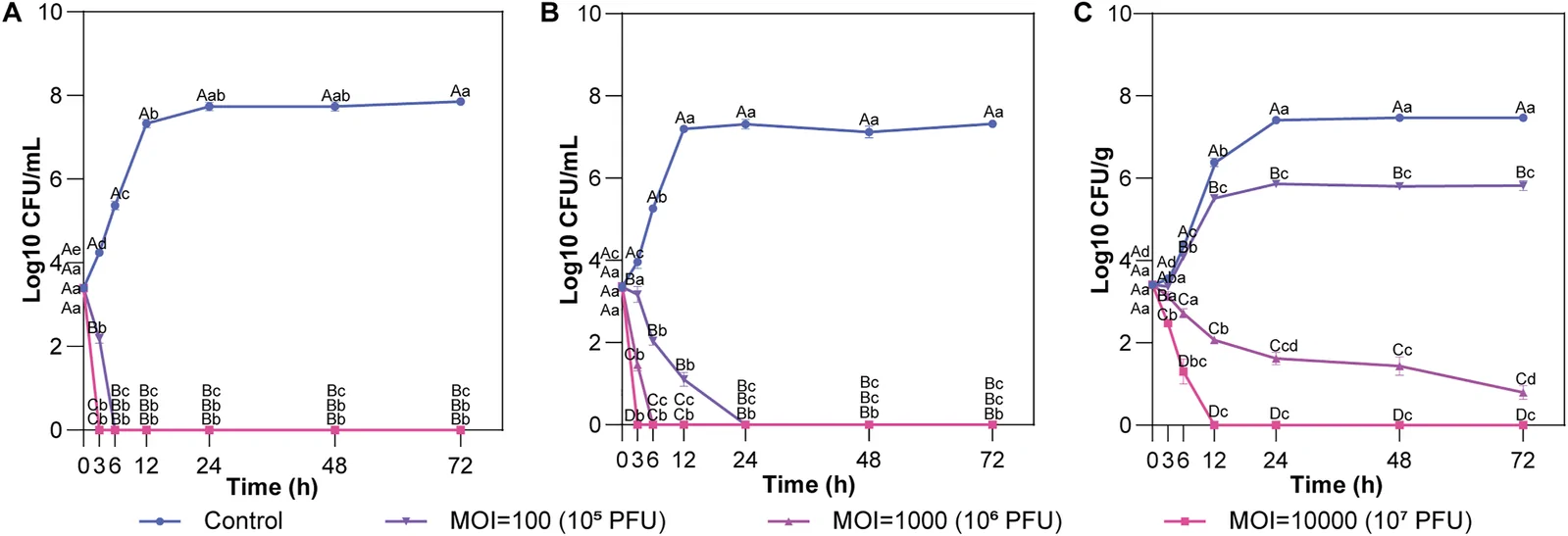
Biocontrol efficacy of phage WAH2 against *B*. *cereus* BY118LC in food matrices at 25 °C. Bacterial counts were monitored in (A) UHT whole milk, (B) rice, and (C) pork samples treated with WAH2 at MOIs of 100, 1000, and 10000. Data are shown as mean ± SD. Different uppercase letters indicate significant differences among different MOI groups at a given time point, while different lowercase letters represent significant differences across different incubation times within the same group (*P* < 0.05).

At 4 °C, the control groups remained relatively stable, but WAH2 still suppressed bacterial growth in a dose-dependent manner (Fig. 8). In milk, counts were reduced to below the detection limit within 12 h at an MOI of 10000 and within 24 h at an MOI of 1000, while an MOI of 100 yielded a 1.14 log_10_ CFU/mL reduction at 72 h (Fig. 8A). In rice, detectable bacteria disappeared by 48 h at an MOI of 10000, and reductions of 1.80 and 0.59 log_10_ CFU/mL were recorded at MOIs of 1000 and 100, respectively, after 72 h (Fig. 8B). In pork, the corresponding reductions at 72 h were 2.12, 0.85, and 0.15 log_10_ CFU/g for MOIs of 10000, 1000, and 100, respectively (Fig. 8C). Again, no bacterial regrowth was detected in any treated group. Collectively, WAH2 effectively suppressed *B. cereus* in all tested food matrices under both 25 °C and 4 °C, supporting its potential as a food biocontrol agent.

**Fig. 8:**
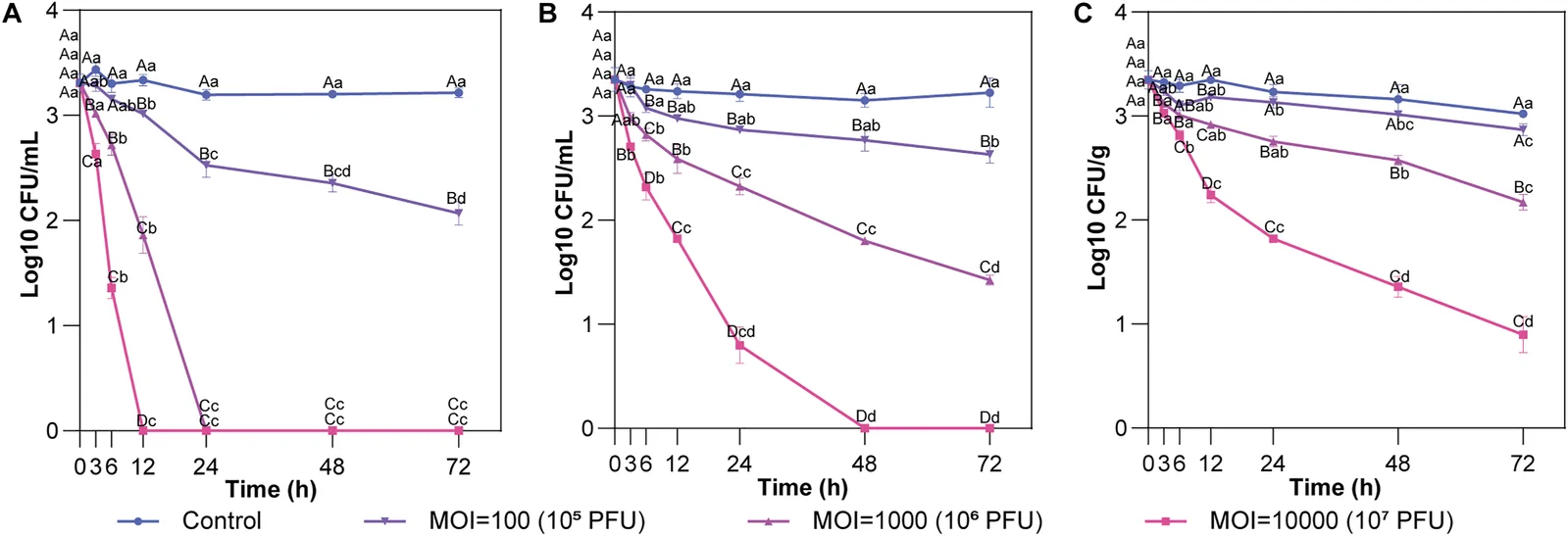
Biocontrol efficacy of phage WAH2 against *B*. *cereus* BY118LC in food matrices at 4 °C. Reduction of bacterial load in (A) UHT whole milk, (B) rice, and (C) pork samples at MOIs of 100, 1000, and 10000. Data are presented as mean ± SD. Statistical differences (*P* < 0.05) are indicated by uppercase letters (comparing different MOIs at the same time interval) and lowercase letters (comparing different time points within the same MOI group).

### 3.8 Phage persistence and resistance in TSB and UHT whole milk

The persistence of WAH2 and the emergence of phage-resistant survivors were evaluated in TSB and UHT whole milk at 25 °C and 4 °C over 120 h. For phage persistence, WAH2 remained highly stable at 4 °C in both matrices, with reductions of only 0.425 and 0.320 log_10_ PFU/mL after 120 h, respectively (Fig. 9A). At 25 °C, the phage titer decreased by 0.808 log_10_ PFU/mL in TSB and 0.677 log_10_ PFU/mL in UHT whole milk over the same period. Infectious phages were still detectable at 120 h under all conditions, indicating that WAH2 persists in these matrices, particularly under refrigeration.

**Fig. 9:**
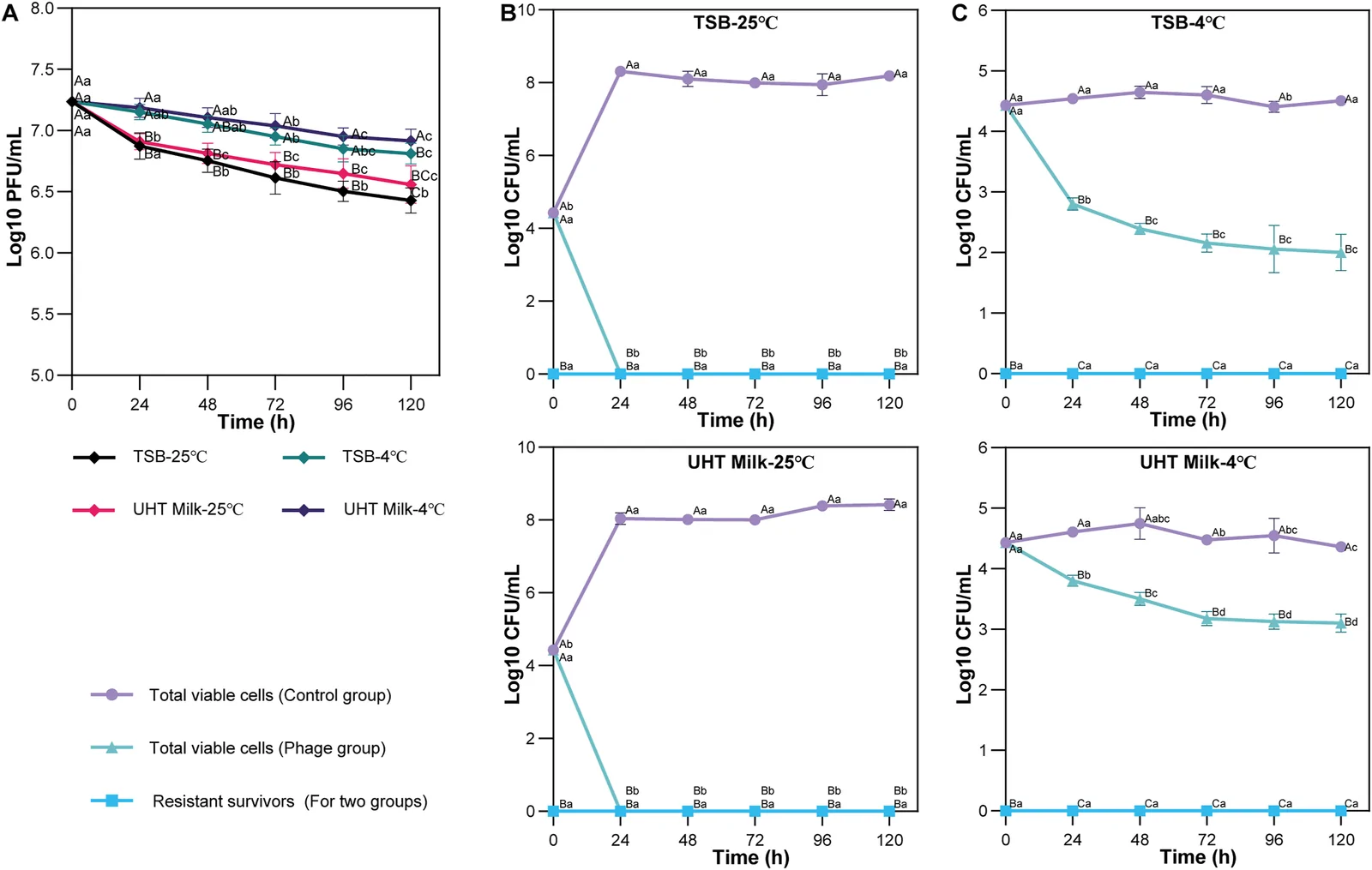
Persistence and resistance of phage WAH2 in TSB and UHT whole milk. (A) The phage titers in TSB and UHT whole milk at 25 °C and 4 °C over 120 h. (B, C) Bacterial survival and phage resistance of *B. cereus* BY118LC incubated with or without WAH2 at 25 °C (B) and 4 °C (C) in TSB and UHT whole milk. Total viable cells were enumerated on TSA without phage: control group (bacteria only) and phage-treated group (bacteria + WAH2). Resistant survivors were enumerated on TSA containing WAH2; no resistant colonies were detected in either group at any time point, and the data are represented by a single line indicating values below the limit of detection. Data are presented as mean ± SD. Statistical differences (*P* < 0.05) are indicated by uppercase letters (comparing different groups at the same time interval) and lowercase letters (comparing different time points within the same group).

For resistance analysis, in the phage-free control groups, bacterial counts at 25 °C increased to approximately 8 log_10_ CFU/mL by 24 h and remaining stable thereafter in both TSB and UHT whole milk (Fig. 9B). At 4 °C, bacterial counts remained relatively stable over 120 h (Fig. 9C). In phage-treated groups, total viable counts were markedly reduced. At 25 °C, no viable bacteria were detected on TSA without phage from 24 h onward in either matrix, with counts below the detection limit (Fig. 9B). At 4 °C, phage-treated groups also showed significantly lower counts than non-phage controls at all time points (Fig. 9C). Notably, no resistant colonies were detected on TSA containing WAH2 for any group at any time point in either matrix or at either temperature, indicating a resistance frequency below the limit of detection (Fig. 9B and C). These results demonstrate that WAH2 does not enrich phage-resistant survivors in TSB or UHT whole milk at either 25 °C or 4 °C.

## 4 Discussion

In this study, WAH2 was identified as a novel lytic *Bacillus* phage and taxonomically assigned to the genus *Bastillevirus* within the family *Herelleviridae*. Genome analysis revealed that WAH2 encodes several tRNA genes, a feature also observed in several related *Bacillus* phages, including DC1/DC2 (Chen et al., 2024), WAH1 (Wan et al., 2026), and Kirov (Kazantseva et al., 2023). Although the functional significance of phage-encoded tRNAs remains incompletely understood, they may reflect adaptation to host translation constraints and contribute to efficient gene expression during infection (Amarillas et al., 2017; Yamik et al., 2026). Notably, no genes associated with lysogeny, virulence, or antibiotic resistance were identified in WAH2, indicating a favorable genomic safety profile. Overall, these findings provide a basis for further evaluation of WAH2 as a candidate for controlling *B. cereus* contamination.

The biological characteristics of phage WAH2 suggest that it is a promising candidate for food biocontrol. WAH2 showed an exceptionally low optimal MOI of 0.0001, consistent with that of the previously reported *Bacillus* phage WAH1 (Wan et al., 2026), indicated that effective infection can be initiated with only a minimal phage dose. This characteristic is advantageous for practical use because it reduces the amount of phage required at the initial stage and thereby lowers production costs. Although WAH2 and WAH1 showed similar optimal MOIs, WAH2 exhibited markedly superior growth kinetics, with a latent period of only 30 min and a burst size of 208 PFU/cell. The rapid adsorption of WAH2 may contribute to its short latent period and large burst size. These results indicate that WAH2 can replicate efficiently within host cells and rapidly amplify progeny phage production, highlighting its potential for rapid suppression of bacterial contamination during food processing. In addition to its favorable growth performance, WAH2 demonstrated strong environmental tolerance, remaining active across a broad range of conditions, including 4–45 °C, pH 5–11, and 2–10% NaCl. This stability range is broader than those reported for vB_BceM-HSE3 (0–40 °C, pH 5–7) (Peng and Yuan, 2018) and Deep-Blue (4–40 °C, pH 5–10) (Hock et al., 2019), suggesting that WAH2 may be better suited to food processing and storage environments, particularly in high-salinity food matrices. Although WAH2 exhibited a relatively narrow host range, lysing only 7 of the 51 tested *B. cereus* strains (13.7%), all susceptible isolates were multidrug-resistant and belonged to distinct sequence types. WAH2 did not lyse representative non-target bacteria, further supporting its narrow lytic spectrum. However, the panel lacked *B. cereus* group members, other foodborne *Bacillus* species, and lactic acid bacteria, which limits a full assessment of its taxonomic specificity and ecological safety. Future studies should include these groups to fully evaluate the safety of WAH2 for food applications. Compared with DZ1 (41.25%, 33/80) (Huang et al., 2024) and WAH1 (64.7%, 33/51) (Wan et al., 2026), WAH2 is more selective; however, this specificity may be advantageous for incorporation into phage cocktail formulations. Given the high genetic diversity of foodborne *B. cereus* (Deng et al., 2021; Hsu et al., 2021; Xiao et al., 2023; Zheng et al., 2024), WAH2 may be best used in combination with other phages to broaden the overall lytic spectrum and improve biocontrol efficacy. Overall, the combination of these favorable traits positions WAH2 as a highly promising candidate for controlling antibiotic-resistant *B. cereus* contamination in the food industry.

The most notable feature of WAH2 is its sustained antibacterial activity. In phage applications, the rapid emergence of phage-resistant mutants is widely recognized as a major challenge (Hatfull et al., 2022; Oromí-Bosch et al., 2023). However, in this study, WAH2 exhibited strong, dose-dependent inhibitory activity across all tested MOIs, and no detectable bacterial regrowth was observed within 36 h. This result contrasts with several recently reported *B. cereus* phages, including DC1/DC2 (Chen et al., 2024), vB_BceP_LY3 (Tan et al., 2024), and Z3 (Li et al., 2024), which all showed regrowth in similar assays. The persistent inhibitory effect of WAH2 suggests that it may have a low tendency to generate resistant mutants. This characteristic is of considerable practical significance, as regrowth caused by resistant bacteria not only compromises antibacterial efficacy but also increases the need for repeated phage application and raises the risk of persistent contamination. The ability of *B. cereus* to form biofilms is a major barrier to effective control in food-processing environments, as biofilm-associated cells are generally more tolerant to disinfectants and heat treatment and may serve as reservoirs for long-term contamination (Huang et al., 2020; Kim et al., 2025a). Food-processing equipment surfaces, especially stainless steel, provide ideal conditions for *B. cereus* attachment and biofilm formation. Previous studies have reported that phages and their derived enzymes can effectively reduce *B. cereus* biofilms (Chen et al., 2024; Camille et al., 2026). In this study, WAH2 exhibited dual activity by inhibiting biofilm formation and disrupting mature biofilms. Mechanistically, WAH2 directly lyses metabolically active cells within biofilms, and its rapid adsorption may facilitate diffusion and infection within the biofilm matrix. Collectively, WAH2 is effective not only against planktonic cells but also against biofilms, highlighting its potential as a promising biocontrol candidate for *B. cereus* contamination.

Spores, due to their high resistance and dormant state, remain a major challenge in food safety control (Leishangthem et al., 2025). Most studies on *B. cereus* phages have focused on vegetative cells, while their effects on spores remains poorly examined (Haridas and Bhat, 2026). Therefore, it is necessary to evaluate the potential of phages in spore control. In this study, phage WAH2 was shown to neither directly inactivate dormant spores nor affect the germination process. Instead, it inhibited the outgrowth of germinated spores under nutrient conditions. This result is consistent with the properties of lytic phages, which rely on metabolically active host cells for infection and replication (Kim, 2025b). In contrast, dormant spores have low metabolic activity and a dense structural barrier, which limits phage action (Kim, 2025b). Once spores germinate and resume metabolic activity in nutrient conditions, they can support phage infection. Notably, viable cells were still detected after heat treatment, suggesting that spore germination in *B. cereus* is heterogeneous (Abee et al., 2011). This finding further highlights the difficulty of spore control and also indicates that a single phage strategy cannot achieve complete eradication. Based on these results, future studies should explore the combined use of germinants and phages to overcome current limitations and improve the potential of phages for spore control.

This study showed that WAH2 can effectively inhibit *B. cereus* in liquid and semi-solid food matrices, it also exhibited strong and sustained antibacterial activity at both room temperature and refrigeration. Overall, it performed better than most reported phages. In previous studies, most phages showed limited efficacy in different food matrices. They often required more time to achieve complete elimination, and bacterial regrowth was common (Chen et al., 2024; Huang et al., 2024; Li et al., 2024). In contrast, WAH2 reduced bacterial count in milk and rice more rapidly. It reduced bacterial count to below the detection limit within a short time and showed no regrowth during the 72 h period. This indicates that WAH2 has good lytic efficiency and sustained antibacterial activity. Notably, WAH2 also remained active in pork, which is a more challenging food matrix. The dense structure of meat and its complex components, such as fat and proteins, may limit phage diffusion or adsorption and reduce contact between phages and host cells (Abdelaziz et al., 2024; Tittarelli et al., 2026; Yao et al., 2025). As a result, the antibacterial effect of many phages in meat is greatly reduced. However, WAH2 still completely eliminated the bacteria at a high MOI, and it also caused a large reduction in bacterial count at lower MOI. This suggests that WAH2 may have broader application potential in complex food systems. Under refrigerated conditions, WAH2 also showed important potential. Previous studies have shown that low temperature can reduce host metabolic activity and weaken phage adsorption and replication (Duc et al., 2020; Yao et al., 2025), which leads to lower antibacterial activity for most phages. However, WAH2 still significantly reduced bacterial count in milk and rice at 4 °C and also showed inhibitory effects in meat. This suggests that WAH2 remains active in cold-chain environments, which is important for food safety control during refrigeration. In summary, WAH2 showed excellent antibacterial activity across different food matrices and temperatures, and no obvious regrowth was observed. These results suggest that WAH2 is a highly promising candidate for food biocontrol.

The sustained antibacterial efficacy of WAH2 in food matrices was further supported by its persistence and resistance profiles. In TSB and UHT whole milk, WAH2 remained infectious for up to 120 h. This sustained infectivity likely contributed to the prolonged suppression of bacterial regrowth observed in the food matrix experiments. In addition, no phage-resistant survivors were detected in TSB or UHT whole milk at either 25 °C or 4 °C, indicating a resistance frequency below the limit of detection. These findings are consistent with the absence of bacterial regrowth observed in the growth inhibition and food matrix experiments. The absence of detectable resistant survivors may be attributed to the use of a conserved host receptor by WAH2 or to a fitness cost associated with resistance. While these results support the potential of WAH2 for sustained biocontrol, resistance evolution under repeated phage exposure and in naturally contaminated foods should be further evaluated. Moreover, only TSB and UHT milk were tested in this study; other food matrices such as rice and meat were not included, and these should be addressed in future work.

Although WAH2 shows promising potential for food biocontrol, its practical application is still limited by several factors. First, WAH2 has a relatively narrow host range, which may limit its coverage of genetically diverse *B. cereus* in naturally contaminated foods. Second, although the phage dose applied in this study is technically feasible, the complexity of food matrices limits uniform delivery, which remains a major obstacle to practical application. Third, the food samples used in this study were pretreated to reduce interference from background microbiota; therefore, the results may not fully reflect biocontrol performance in real food environments. To address these limitations, future research can proceed in following directions: constructing phage cocktails based on different receptor-binding proteins or complementary host spectra to broaden the lytic spectrum; modifying phage tail fiber proteins through site-directed mutagenesis, domain swapping, or directed evolution to expand the host range; and encapsulating phages or their encoded lytic enzymes with food-grade materials such as alginate and chitosan to improve stability, reduce required dose, and enhance delivery. All these strategies require systematic validation under actual processing and storage conditions to enhance the practical application value of WAH2 in food biocontrol.

## 5 Conclusion

This study isolated and characterized a novel lytic phage, WAH2, against *B. cereus*. WAH2 had a relatively narrow host range but showed strong environmental tolerance and replication ability. Genome analysis classified WAH2 into the genus *Bastillevirus* within the family *Herelleviridae*. No antibiotic resistance genes, virulence genes, or lysogeny-related elements were detected, indicating high biosafety. WAH2 showed strong lytic activity, with no bacterial regrowth within 36 h. It also significantly inhibited biofilm formation and disrupted mature biofilms. In addition, WAH2 could neither directly inactivate dormant spores nor affect spore germination, but it significantly inhibited the outgrowth of germinated spores. Notably, WAH2 effectively suppressed the growth of *B. cereus* in milk, rice, and pork at both 25 and 4 °C, and no regrowth was observed. In TSB and milk, WAH2 persisted for 120 h without detectable resistant survivors. In summary, WAH2 is a safe and effective biocontrol candidate with good application potential for controlling *B. cereus* contamination in food.

## CRediT authorship contribution statement

Peng Wan: Writing–original draft, Methodology, Investigation, Data curation, Visualization. Zhengyuan He: Writing–review & editing, Methodology, Investigation. Weiqi Liu: Resources, Methodology. Honghao Huang: Visualization, Investigation. Huan Chen: Methodology. Sihan Lin: Methodology. Wenguang Xiong: Writing–review & editing. Zhenling Zeng: Writing–review & editing, Funding acquisition, Supervision, Conceptualization.

## Declaration of generative AI and AI-assisted technologies in the writing process

During the preparation of this manuscript, the authors used Grammarly and Gemini to help identify and correct grammatical errors. Following the use of these tools, the authors thoroughly reviewed and revised the content as necessary and assume full responsibility for the final published work.

## Funding

This work was supported by Guangdong Major Project of Basic and Applied Basic Research (Grant No. 2020B0301030005).

## Declaration of competing interest

The authors declare that they have no known competing financial interests or personal relationships that could have appeared to influence the work reported in this paper.
