# Supplementary material for: A novel lytic phage with potent efficacy against Bacillus cereus, its biofilms and spores, and biocontrol potential in food matrices
Source: Curr Res Food Sci. 2026 Oct 19;13:101603. doi: 10.1016/j.crfs.2026.101603 (PMC13717040; doi:10.1016/j.crfs.2026.101603)
Supplement: Supplementary file 1 [file mmc1.docx]

**Table S1** Host range of phage WAH2 against non-target strains.

| Bacteria | | ST^a^ | Spot assay^b^ | Source/Reference^c^ |
| --- | --- | --- | --- | --- |
| Gram-positive bacteria | | | |  |
| *Staphylococcus aureus* | RN 4220 | / | - | Laboratory Stock |
|  | ATCC 29213 | / | - | ATCC |
|  | ATCC 43300 | / | - | ATCC |
|  | GDM7P049A | 1 | - | (1) |
|  | GDC6P036P | 9 | - | (1) |
|  | GDC6P090P | 398 | - | (1) |
| *Staphylococcus borealis* | GDA23P27T | 64 | - | (2) |
|  | GDA23P42T | 64 | - | (2) |
|  | GDA23P288T | 190 | - | (2) |
|  | GXK23P15W6 | / | - | (3) |
|  | GXK23P21W20 | / | - | (3) |
| *Enterococcus faecalis* | ATCC 29212 | / | - | ATCC |
|  | GD22P364 | 506 | - | (4) |
|  | GD23P61 | 16 | - | (4) |
|  | GD24P32 | 591 | - | (4) |
| *Enterococcus faecium* | GD23P211 | / | - | (4) |
| *Clostridium perfringens*^d^ | ATCC 13124 | / | - | ATCC |
|  | GDZ21C61W | / | - | (5) |
| Gram-negative bacteria |  |  |  |  |
| *Escherichia coli* | ATCC 25922 | / | - | ATCC |
|  | SHP 45 | / | - | (6) |
|  | GP53 | / | - | (7) |
| *Salmonella Typhimurium* | ATCC 14028 | / | - | ATCC |
|  | 26FS14 | / | - | (8) |
|  | 26FS26 | / | - | (8) |
|  | S235 | / | - | (8) |
|  | S226 | / | - | (8) |

^a^ “ST”, the abbreviation of sequence type; “/”, ST not available.

^b^ “-”, no lysis.

^c^ “ATCC”, the abbreviation of American Type Culture Collection.

^d^ *C. perfringens* was tested under anaerobic conditions.

**Reference**

(1) Li W, Xiong W, Guo J, Sun N, Zeng Z. Prevalence of the *cfr* gene and co-dissemination with the *fosD* gene in swine-derived *Staphylococcus aureus*. J Glob Antimicrob Resist. 2026;46:57-62.

(2) Huang H, Zhao F, Zhao R, Diao L, Wan P, He Z, Chen H, Zhang L, Li Y, Zeng Z. Tigecycline-resistant *Staphylococcus* in waiting pens of a pig slaughterhouse: genomic insights into a food safety alert. J Antimicrob Chemother. 2026;81(10):dkag254.

(3) Huang H, Wan P, Zhao R, Hu J, Zhang L, Xiong W, Chen Y, Chen Y, Zeng Z. Emerging zoonotic pathogen *Staphylococcus borealis* mediates longitudinal increase and environmental contamination of tigecycline resistance throughout the pig production period. J Hazard Mater. 2025;497:139606.

(4) Hu J, Yang J, Zeng Z. Detection and characterization of oxazolidinone resistance genes in *enterococci* from companion animals. One Health Advances. 2026;4(1).

(5) Zhang W, Lu Y, Ma M, Yang J, Huang H, Peng X, Zeng Z, Zeng D. *Ex vivo* pharmacokinetic/pharmacodynamic of hexahydrocolupulone against *Clostridium perfringens* in broiler chickens. Front Vet Sci. 2024;11:1362292.

(6) Liu YY, Wang Y, Walsh TR, Yi LX, Zhang R, Spencer J, Doi Y, Tian G, Dong B, Huang X, Yu LF, Gu D, Ren H, Chen X, Lv L, He D, Zhou H, Liang Z, Liu JH, Shen J. Emergence of plasmid-mediated colistin resistance mechanism MCR-1 in animals and human beings in China: a microbiological and molecular biological study. Lancet Infect Dis. 2016;16(2):161-8.

(7) Zhao R, Wan P, Huang H, Li Q, Zeng Z, Xiong W. Harnessing CRISPR-Cas9 and CRISPRi systems to reverse antibiotic resistance in a clinical multidrug-resistant *Escherichia coli* isolate. J Antimicrob Chemother. 2026;81(1):dkaf442.

(8) Kong L, Lu Y, Yang L, Zhang W, Zuo B, Peng X, Qin Z, Li M, Zeng Z, Zeng D. Pharmacokinetics and Pharmacodynamics of Colistin Combined With Isopropoxy Benzene Guanidine Against *mcr-1*-Positive *Salmonella* in an Intestinal Infection Model. Front Microbiol. 2022;13:907116.

**Table S2** The predicted functional ORFs of phage WAH2.

| ORFs | Start | Stop | Strand | Product | Function Classification |
| --- | --- | --- | --- | --- | --- |
| WAH2_1 | 19 | 666 | + | FtsK/SpoIIIE-like protein | unsorted |
| WAH2_2 | 837 | 1517 | + | hypothetical protein | hypothetical protein |
| WAH2_3 | 1569 | 1736 | + | hypothetical protein | hypothetical protein |
| WAH2_4 | 1767 | 2066 | + | hypothetical protein | hypothetical protein |
| WAH2_5 | 2080 | 2553 | + | hypothetical protein | hypothetical protein |
| WAH2_6 | 2637 | 2951 | + | hypothetical protein | hypothetical protein |
| WAH2_7 | 2958 | 3350 | + | hypothetical protein | hypothetical protein |
| WAH2_8 | 3377 | 3550 | + | hypothetical protein | hypothetical protein |
| WAH2_9 | 3580 | 4002 | + | hypothetical protein | hypothetical protein |
| WAH2_10 | 4017 | 4886 | + | hypothetical protein | hypothetical protein |
| WAH2_11 | 4899 | 5309 | + | hypothetical protein | hypothetical protein |
| WAH2_12 | 5302 | 5634 | + | hypothetical protein | hypothetical protein |
| WAH2_13 | 5785 | 5964 | + | hypothetical protein | hypothetical protein |
| WAH2_14 | 5979 | 6179 | + | hypothetical protein | hypothetical protein |
| WAH2_15 | 6176 | 6346 | + | hypothetical protein | hypothetical protein |
| WAH2_16 | 6359 | 6571 | + | hypothetical protein | hypothetical protein |
| WAH2_17 | 6571 | 6795 | + | hypothetical protein | hypothetical protein |
| WAH2_18 | 6795 | 7145 | + | hypothetical protein | hypothetical protein |
| WAH2_19 | 7135 | 7419 | + | hypothetical protein | hypothetical protein |
| WAH2_20 | 7429 | 7779 | + | hypothetical protein | hypothetical protein |
| WAH2_21 | 7796 | 8722 | + | RNA ligase | replication and regulation |
| WAH2_22 | 8844 | 9056 | + | hypothetical protein | hypothetical protein |
| WAH2_23 | 9069 | 9311 | + | hypothetical protein | hypothetical protein |
| WAH2_24 | 9304 | 9507 | + | hypothetical protein | hypothetical protein |
| WAH2_25 | 9518 | 9667 | + | putative ligase | replication and regulation |
| WAH2_26 | 9664 | 9996 | + | hypothetical protein | hypothetical protein |
| WAH2_27 | 10017 | 10241 | + | hypothetical protein | hypothetical protein |
| WAH2_28 | 10241 | 10528 | + | hypothetical protein | hypothetical protein |
| WAH2_29 | 10528 | 10803 | + | hypothetical protein | hypothetical protein |
| WAH2_30 | 10927 | 11205 | + | hypothetical protein | hypothetical protein |
| WAH2_31 | 11234 | 11443 | + | hypothetical protein | hypothetical protein |
| WAH2_32 | 11468 | 11881 | + | membrane protein | structure and assembly |
| WAH2_33 | 12241 | 12462 | + | hypothetical protein | hypothetical protein |
| WAH2_34 | 12561 | 12875 | + | hypothetical protein | hypothetical protein |
| WAH2_35 | 13047 | 13937 | + | hypothetical protein | hypothetical protein |
| WAH2_36 | 13941 | 14210 | + | hypothetical protein | hypothetical protein |
| WAH2_37 | 14295 | 14540 | + | hypothetical protein | hypothetical protein |
| WAH2_38 | 14540 | 14740 | + | hypothetical protein | hypothetical protein |
| WAH2_39 | 14740 | 14958 | + | hypothetical protein | hypothetical protein |
| WAH2_40 | 14989 | 15153 | + | hypothetical protein | hypothetical protein |
| WAH2_41 | 15284 | 15655 | + | HTH DNA binding protein | replication and regulation |
| WAH2_42 | 15724 | 15990 | + | hypothetical protein | hypothetical protein |
| WAH2_43 | 15990 | 16142 | + | hypothetical protein | hypothetical protein |
| WAH2_44 | 16132 | 16347 | + | hypothetical protein | hypothetical protein |
| WAH2_45 | 16418 | 16774 | + | hypothetical protein | hypothetical protein |
| WAH2_46 | 16771 | 17139 | + | hypothetical protein | hypothetical protein |
| WAH2_47 | 17209 | 17448 | + | hypothetical protein | hypothetical protein |
| WAH2_48 | 17448 | 17645 | + | hypothetical protein | hypothetical protein |
| WAH2_49 | 17638 | 17850 | + | hypothetical protein | hypothetical protein |
| WAH2_50 | 17850 | 18020 | + | hypothetical protein | hypothetical protein |
| WAH2_51 | 18014 | 18163 | + | hypothetical protein | hypothetical protein |
| WAH2_52 | 18216 | 18401 | + | hypothetical protein | hypothetical protein |
| WAH2_53 | 18433 | 18648 | + | hypothetical protein | hypothetical protein |
| WAH2_54 | 18718 | 18954 | + | hypothetical protein | hypothetical protein |
| WAH2_55 | 19016 | 19351 | + | hypothetical protein | hypothetical protein |
| WAH2_56 | 19353 | 19709 | + | hypothetical protein | hypothetical protein |
| WAH2_57 | 19702 | 19938 | + | hypothetical protein | hypothetical protein |
| WAH2_58 | 20050 | 20379 | + | hypothetical protein | hypothetical protein |
| WAH2_59 | 20396 | 20599 | + | hypothetical protein | hypothetical protein |
| WAH2_60 | 20589 | 20807 | + | hypothetical protein | hypothetical protein |
| WAH2_61 | 20823 | 21080 | + | hypothetical protein | hypothetical protein |
| WAH2_62 | 21080 | 21289 | + | hypothetical protein | hypothetical protein |
| WAH2_63 | 22795 | 23289 | + | hypothetical protein | hypothetical protein |
| WAH2_64 | 23333 | 23686 | + | hypothetical protein | hypothetical protein |
| WAH2_65 | 24362 | 24559 | - | hypothetical protein | hypothetical protein |
| WAH2_66 | 24586 | 24750 | - | hypothetical protein | hypothetical protein |
| WAH2_67 | 24752 | 24904 | - | hypothetical protein | hypothetical protein |
| WAH2_68 | 24906 | 25613 | - | hypothetical protein | hypothetical protein |
| WAH2_69 | 25626 | 26279 | - | hypothetical protein | hypothetical protein |
| WAH2_70 | 26292 | 26456 | - | hypothetical protein | hypothetical protein |
| WAH2_71 | 26468 | 26908 | - | hypothetical protein | hypothetical protein |
| WAH2_72 | 26921 | 27154 | - | hypothetical protein | hypothetical protein |
| WAH2_73 | 27151 | 27390 | - | hypothetical protein | hypothetical protein |
| WAH2_74 | 27350 | 27595 | - | hypothetical protein | hypothetical protein |
| WAH2_75 | 27600 | 27779 | - | hypothetical protein | hypothetical protein |
| WAH2_76 | 27779 | 28000 | - | hypothetical protein | hypothetical protein |
| WAH2_77 | 28012 | 28191 | - | hypothetical protein | hypothetical protein |
| WAH2_78 | 28188 | 28493 | - | hypothetical protein | hypothetical protein |
| WAH2_79 | 28508 | 28780 | - | hypothetical protein | hypothetical protein |
| WAH2_80 | 28847 | 30034 | - | tRNA splicing ligase | replication and regulation |
| WAH2_81 | 30104 | 30376 | - | hypothetical protein | hypothetical protein |
| WAH2_82 | 30373 | 30648 | - | hypothetical protein | hypothetical protein |
| WAH2_83 | 30846 | 31016 | - | hypothetical protein | hypothetical protein |
| WAH2_84 | 31007 | 31240 | - | hypothetical protein | hypothetical protein |
| WAH2_85 | 31251 | 31457 | - | hypothetical protein | hypothetical protein |
| WAH2_86 | 31570 | 31773 | - | hypothetical protein | hypothetical protein |
| WAH2_87 | 31770 | 32144 | - | hypothetical protein | hypothetical protein |
| WAH2_88 | 32149 | 32370 | - | hypothetical protein | hypothetical protein |
| WAH2_89 | 32367 | 32762 | - | hypothetical protein | hypothetical protein |
| WAH2_90 | 32759 | 33154 | - | hypothetical protein | hypothetical protein |
| WAH2_91 | 33234 | 33617 | - | hypothetical protein | hypothetical protein |
| WAH2_92 | 33614 | 33817 | - | hypothetical protein | hypothetical protein |
| WAH2_93 | 33836 | 34597 | - | hypothetical protein | hypothetical protein |
| WAH2_94 | 34581 | 34781 | - | hypothetical protein | hypothetical protein |
| WAH2_95 | 34759 | 35034 | - | hypothetical protein | hypothetical protein |
| WAH2_96 | 35031 | 35276 | - | hypothetical protein | hypothetical protein |
| WAH2_97 | 35289 | 35483 | - | hypothetical protein | hypothetical protein |
| WAH2_98 | 35486 | 36064 | - | hypothetical protein | hypothetical protein |
| WAH2_99 | 36066 | 36242 | - | hypothetical protein | hypothetical protein |
| WAH2_100 | 36239 | 36856 | - | hypothetical protein | hypothetical protein |
| WAH2_101 | 36880 | 37053 | - | hypothetical protein | hypothetical protein |
| WAH2_102 | 37119 | 37319 | - | hypothetical protein | hypothetical protein |
| WAH2_103 | 37312 | 37974 | - | hypothetical protein | hypothetical protein |
| WAH2_104 | 37975 | 38298 | - | replication initiation protein | replication and regulation |
| WAH2_105 | 38364 | 38864 | - | dihydrofolate reductase | replication and regulation |
| WAH2_106 | 38943 | 39311 | - | hypothetical protein | hypothetical protein |
| WAH2_107 | 39314 | 39583 | - | hypothetical protein | hypothetical protein |
| WAH2_108 | 39584 | 39802 | - | hypothetical protein | hypothetical protein |
| WAH2_109 | 39792 | 40394 | - | kinase | unsorted |
| WAH2_110 | 40387 | 40554 | - | hypothetical protein | hypothetical protein |
| WAH2_111 | 40551 | 41435 | - | thymidylate synthase | replication and regulation |
| WAH2_112 | 41578 | 41769 | - | hypothetical protein | hypothetical protein |
| WAH2_113 | 41766 | 42005 | - | ribosome associated inhibitor A; zinc finger domain | replication and regulation |
| WAH2_114 | 42062 | 43459 | - | hypothetical protein | hypothetical protein |
| WAH2_115 | 43538 | 44386 | - | hypothetical protein | hypothetical protein |
| WAH2_116 | 44405 | 44740 | - | hypothetical protein | hypothetical protein |
| WAH2_117 | 44737 | 45522 | - | hypothetical protein | hypothetical protein |
| WAH2_118 | 45488 | 45700 | - | hypothetical protein | hypothetical protein |
| WAH2_119 | 45746 | 46042 | - | hypothetical protein | hypothetical protein |
| WAH2_120 | 46042 | 46311 | - | hypothetical protein | hypothetical protein |
| WAH2_121 | 46311 | 46487 | - | hypothetical protein | hypothetical protein |
| WAH2_122 | 46582 | 47676 | - | endolysin | lysis |
| WAH2_123 | 47704 | 48153 | - | hypothetical protein | hypothetical protein |
| WAH2_124 | 48200 | 49996 | - | terminase large subunit | packaging |
| WAH2_125 | 49996 | 50415 | - | terminase small subunit | packaging |
| WAH2_126 | 50402 | 50689 | - | hypothetical protein | hypothetical protein |
| WAH2_127 | 50686 | 51180 | - | hypothetical protein | hypothetical protein |
| WAH2_128 | 51182 | 51466 | - | membrane protein | structure and assembly |
| WAH2_129 | 51586 | 51897 | + | membrane protein | structure and assembly |
| WAH2_130 | 51872 | 52225 | + | hypothetical protein | hypothetical protein |
| WAH2_131 | 52244 | 52561 | + | hemolysin | lysis |
| WAH2_132 | 52578 | 52811 | + | hypothetical protein | hypothetical protein |
| WAH2_133 | 52851 | 53099 | - | transcriptional regulator | replication and regulation |
| WAH2_134 | 54463 | 55299 | + | tail spike protein | structure and assembly |
| WAH2_135 | 55606 | 55872 | + | portal protein | structure and assembly |
| WAH2_136 | 55911 | 57584 | + | portal protein | structure and assembly |
| WAH2_137 | 57553 | 57795 | + | hypothetical protein | hypothetical protein |
| WAH2_138 | 57875 | 58687 | + | prohead protease | structure and assembly |
| WAH2_139 | 58687 | 59613 | + | hypothetical protein | hypothetical protein |
| WAH2_140 | 59834 | 61273 | + | major head protein | structure and assembly |
| WAH2_141 | 61513 | 61734 | + | hypothetical protein | hypothetical protein |
| WAH2_142 | 61744 | 62625 | + | tail fiber protein | structure and assembly |
| WAH2_143 | 62637 | 63422 | + | virion structural protein | structure and assembly |
| WAH2_144 | 63422 | 64042 | + | hypothetical protein | hypothetical protein |
| WAH2_145 | 64057 | 64935 | + | hypothetical protein | hypothetical protein |
| WAH2_146 | 64948 | 65160 | + | hypothetical protein | hypothetical protein |
| WAH2_147 | 65186 | 66898 | + | tail sheath protein | structure and assembly |
| WAH2_148 | 66949 | 67377 | + | virion structural protein | structure and assembly |
| WAH2_149 | 67559 | 68221 | + | L-alanyl-D-glutamate peptidas | lysis |
| WAH2_150 | 68323 | 68754 | + | tail assembly chaperone | structure and assembly |
| WAH2_151 | 68891 | 69382 | + | tail assembly chaperone | structure and assembly |
| WAH2_152 | 69437 | 73315 | + | putative tail lysin protein | lysis |
| WAH2_153 | 73315 | 73566 | + | hypothetical protein | hypothetical protein |
| WAH2_154 | 73585 | 75831 | + | tail protein with lysin activity | lysis |
| WAH2_155 | 75867 | 81962 | + | tail fiber protein | structure and assembly |
| WAH2_156 | 81975 | 84584 | + | minor tail protein | structure and assembly |
| WAH2_157 | 84571 | 84990 | + | hypothetical protein | hypothetical protein |
| WAH2_158 | 84987 | 85484 | + | tail fiber protein | structure and assembly |
| WAH2_159 | 85541 | 85756 | + | hypothetical protein | hypothetical protein |
| WAH2_160 | 85843 | 86628 | + | virion structural protein | structure and assembly |
| WAH2_161 | 86630 | 87166 | + | hypothetical protein | hypothetical protein |
| WAH2_162 | 87166 | 87885 | + | baseplate protein | structure and assembly |
| WAH2_163 | 87897 | 88943 | + | baseplate J-like protein | structure and assembly |
| WAH2_164 | 88954 | 91449 | + | virion structural protein | structure and assembly |
| WAH2_165 | 91461 | 95003 | + | tail protein | structure and assembly |
| WAH2_166 | 95017 | 95211 | + | hypothetical protein | hypothetical protein |
| WAH2_167 | 95287 | 97059 | + | DNA helicase | replication and regulation |
| WAH2_168 | 97146 | 97982 | + | HTH DNA binding protein | replication and regulation |
| WAH2_169 | 98061 | 99845 | + | HTH DNA binding protein | replication and regulation |
| WAH2_170 | 99880 | 101370 | + | DNA helicase | replication and regulation |
| WAH2_171 | 101409 | 101669 | + | hypothetical protein | hypothetical protein |
| WAH2_172 | 101669 | 101911 | + | hypothetical protein | hypothetical protein |
| WAH2_173 | 101911 | 102138 | + | hypothetical protein | hypothetical protein |
| WAH2_174 | 102135 | 103229 | + | exonuclease II | replication and regulation |
| WAH2_175 | 103233 | 103820 | + | exonuclease I | replication and regulation |
| WAH2_176 | 103908 | 105806 | + | exonuclease | replication and regulation |
| WAH2_177 | 105793 | 106404 | + | anti-sigma factor | replication and regulation |
| WAH2_178 | 106416 | 107498 | + | DNA primase | replication and regulation |
| WAH2_179 | 107508 | 107633 | + | hypothetical protein | hypothetical protein |
| WAH2_180 | 107704 | 108600 | + | dUTP nucleotidohydrolase | replication and regulation |
| WAH2_181 | 108597 | 108866 | + | hypothetical protein | hypothetical protein |
| WAH2_182 | 108879 | 109142 | + | hypothetical protein | hypothetical protein |
| WAH2_183 | 109132 | 109419 | + | hypothetical protein | hypothetical protein |
| WAH2_184 | 109438 | 109848 | + | hypothetical protein | hypothetical protein |
| WAH2_185 | 109874 | 110068 | + | hypothetical protein | hypothetical protein |
| WAH2_186 | 110069 | 110632 | + | RusA-like Holliday junction resolvase | replication and regulation |
| WAH2_187 | 110683 | 113004 | + | ribonucleotide reductase large subunit | replication and regulation |
| WAH2_188 | 113118 | 113390 | + | hypothetical protein | hypothetical protein |
| WAH2_189 | 113392 | 113670 | + | hypothetical protein | hypothetical protein |
| WAH2_190 | 113639 | 113914 | + | hypothetical protein | hypothetical protein |
| WAH2_191 | 113907 | 114119 | + | hypothetical protein | hypothetical protein |
| WAH2_192 | 114129 | 115253 | + | ribonucleoside-diphosphate reductase | replication and regulation |
| WAH2_193 | 115237 | 115689 | + | flavodoxin | replication and regulation |
| WAH2_194 | 115694 | 116158 | + | hypothetical protein | hypothetical protein |
| WAH2_195 | 116164 | 116433 | + | thioredoxin | replication and regulation |
| WAH2_196 | 116433 | 116633 | + | hypothetical protein | hypothetical protein |
| WAH2_197 | 116630 | 117037 | + | hypothetical protein | hypothetical protein |
| WAH2_198 | 117037 | 117507 | + | hypothetical protein | hypothetical protein |
| WAH2_199 | 117488 | 117712 | + | hypothetical protein | hypothetical protein |
| WAH2_200 | 117712 | 117876 | + | hypothetical protein | hypothetical protein |
| WAH2_201 | 117960 | 118730 | + | hypothetical protein | hypothetical protein |
| WAH2_202 | 118732 | 119076 | + | DNA binding protein | replication and regulation |
| WAH2_203 | 119039 | 119275 | + | hypothetical protein | hypothetical protein |
| WAH2_204 | 119360 | 119566 | + | hypothetical protein | hypothetical protein |
| WAH2_205 | 119550 | 119735 | + | hypothetical protein | hypothetical protein |
| WAH2_206 | 119732 | 120007 | + | hypothetical protein | hypothetical protein |
| WAH2_207 | 120019 | 120216 | + | hypothetical protein | hypothetical protein |
| WAH2_208 | 120230 | 120466 | + | hypothetical protein | hypothetical protein |
| WAH2_209 | 120468 | 120914 | + | hypothetical protein | hypothetical protein |
| WAH2_210 | 120916 | 121278 | + | hypothetical protein | hypothetical protein |
| WAH2_211 | 121253 | 121447 | + | hypothetical protein | hypothetical protein |
| WAH2_212 | 121449 | 121925 | + | hypothetical protein | hypothetical protein |
| WAH2_213 | 121927 | 122127 | + | hypothetical protein | hypothetical protein |
| WAH2_214 | 122201 | 124330 | + | DNA polymerase | replication and regulation |
| WAH2_215 | 124560 | 125555 | + | DNA polymerase II | replication and regulation |
| WAH2_216 | 125565 | 125762 | + | hypothetical protein | hypothetical protein |
| WAH2_217 | 125807 | 126088 | + | hypothetical protein | hypothetical protein |
| WAH2_218 | 126064 | 126339 | + | hypothetical protein | hypothetical protein |
| WAH2_219 | 126326 | 126616 | + | hypothetical protein | hypothetical protein |
| WAH2_220 | 126603 | 126968 | + | hypothetical protein | hypothetical protein |
| WAH2_221 | 126972 | 127229 | + | hypothetical protein | hypothetical protein |
| WAH2_222 | 127302 | 127664 | + | hypothetical protein | hypothetical protein |
| WAH2_223 | 127664 | 127936 | + | hypothetical protein | hypothetical protein |
| WAH2_224 | 127933 | 128244 | + | hypothetical protein | hypothetical protein |
| WAH2_225 | 128244 | 128408 | + | hypothetical protein | hypothetical protein |
| WAH2_226 | 128398 | 128685 | + | hypothetical protein | hypothetical protein |
| WAH2_227 | 128746 | 128964 | + | hypothetical protein | hypothetical protein |
| WAH2_228 | 128964 | 129182 | + | hypothetical protein | hypothetical protein |
| WAH2_229 | 129193 | 129720 | + | hypothetical protein | hypothetical protein |
| WAH2_230 | 129720 | 129923 | + | hypothetical protein | hypothetical protein |
| WAH2_231 | 129920 | 130096 | + | hypothetical protein | hypothetical protein |
| WAH2_232 | 130109 | 130402 | + | hypothetical protein | hypothetical protein |
| WAH2_233 | 130494 | 130682 | + | hypothetical protein | hypothetical protein |
| WAH2_234 | 130669 | 131964 | + | single strand DNA binding protein | replication and regulation |
| WAH2_235 | 132034 | 133233 | + | UvsX-like recombinase | replication and regulation |
| WAH2_236 | 133283 | 133636 | + | hypothetical protein | hypothetical protein |
| WAH2_237 | 133659 | 134303 | + | RNA polymerase sigma factor | replication and regulation |
| WAH2_238 | 134300 | 134446 | + | hypothetical protein | hypothetical protein |
| WAH2_239 | 134503 | 134805 | + | holin | lysis |
| WAH2_240 | 134805 | 135284 | + | Ig domain containing protein | unsorted |
| WAH2_241 | 135399 | 135884 | + | hypothetical protein | hypothetical protein |
| WAH2_242 | 135884 | 136864 | + | exopolyphosphatase | unsorted |
| WAH2_243 | 136851 | 137177 | + | hypothetical protein | hypothetical protein |
| WAH2_244 | 137179 | 138465 | + | hypothetical protein | hypothetical protein |
| WAH2_245 | 138490 | 138858 | + | hypothetical protein | hypothetical protein |
| WAH2_246 | 138870 | 139256 | + | hypothetical protein | hypothetical protein |
| WAH2_247 | 139332 | 139565 | + | hypothetical protein | hypothetical protein |
| WAH2_248 | 139575 | 140198 | + | hypothetical protein | hypothetical protein |
| WAH2_249 | 140253 | 140426 | + | hypothetical protein | hypothetical protein |
| WAH2_250 | 140414 | 140794 | + | hypothetical protein | hypothetical protein |
| WAH2_251 | 140791 | 141513 | + | hypothetical protein | hypothetical protein |
| WAH2_252 | 141515 | 142084 | + | carboxypeptidase | lysis |
| WAH2_253 | 142086 | 142205 | + | hypothetical protein | hypothetical protein |
| WAH2_254 | 142202 | 142417 | + | hypothetical protein | hypothetical protein |
| WAH2_255 | 142407 | 142913 | + | hypothetical protein | hypothetical protein |
| WAH2_256 | 142879 | 143742 | + | hypothetical protein | hypothetical protein |
| WAH2_257 | 144066 | 144761 | + | hypothetical protein | hypothetical protein |
| WAH2_258 | 144773 | 145276 | + | virion structural protein | structure and assembly |
| WAH2_259 | 145362 | 145592 | + | hypothetical protein | hypothetical protein |
| WAH2_260 | 145608 | 146198 | + | DNA binding protein | replication and regulation |
| WAH2_261 | 146261 | 146491 | + | hypothetical protein | hypothetical protein |
| WAH2_262 | 146491 | 146754 | + | hypothetical protein | hypothetical protein |
| WAH2_263 | 146754 | 146918 | + | hypothetical protein | hypothetical protein |
| WAH2_264 | 146915 | 147238 | + | hypothetical protein | hypothetical protein |
| WAH2_265 | 147274 | 148005 | + | hypothetical protein | hypothetical protein |
| WAH2_266 | 148095 | 149012 | + | RNA polymerase sigma factor | replication and regulation |
| WAH2_267 | 149095 | 149283 | + | hypothetical protein | hypothetical protein |
| WAH2_268 | 149287 | 149676 | + | hypothetical protein | hypothetical protein |
| WAH2_269 | 149750 | 149944 | + | hypothetical protein | hypothetical protein |
| WAH2_270 | 149928 | 150287 | + | hypothetical protein | hypothetical protein |
| WAH2_271 | 150284 | 150394 | + | hypothetical protein | hypothetical protein |
| WAH2_272 | 150398 | 150718 | + | hypothetical protein | hypothetical protein |
| WAH2_273 | 150779 | 151504 | + | metal-dependent hydrolase | unsorted |
| WAH2_274 | 151517 | 151696 | + | hypothetical protein | hypothetical protein |
| WAH2_275 | 151698 | 152078 | + | hypothetical protein | hypothetical protein |
| WAH2_276 | 152068 | 153222 | + | hypothetical protein | hypothetical protein |
| WAH2_277 | 153224 | 153643 | + | hypothetical protein | hypothetical protein |
| WAH2_278 | 153785 | 154858 | + | partition protein | structure and assembly |
| WAH2_279 | 154864 | 155142 | + | hypothetical protein | hypothetical protein |
| WAH2_280 | 155227 | 155388 | - | hypothetical protein | hypothetical protein |
| WAH2_281 | 155466 | 155654 | + | hypothetical protein | hypothetical protein |
| WAH2_282 | 156072 | 157292 | + | hypothetical protein | hypothetical protein |
| WAH2_283 | 157305 | 157418 | + | hypothetical protein | hypothetical protein |
| WAH2_284 | 157408 | 158991 | + | DNA translocase | unsorted |

**Table S3** Genomic homology alignment of phage WAH2.

| Description | Max Score | Total Score | Query Cover | E value | Per. ident | Length | Accession |
| --- | --- | --- | --- | --- | --- | --- | --- |
| *Bacillus* phage Kamfam | 25490 | 1.68E+05 | 92% | 0 | 92.47 | 161830 | [MH638310.1](https://www.ncbi.nlm.nih.gov/nucleotide/MH638310.1?report=genbank&log$=nucltop&blast_rank=1&RID=VJAMK0N9016) |
| *Bacillus* phage OTooleKemple52 | 25490 | 1.68E+05 | 92% | 0 | 92.47 | 161807 | [MF288921.1](https://www.ncbi.nlm.nih.gov/nucleotide/MF288921.1?report=genbank&log$=nucltop&blast_rank=2&RID=VJAMK0N9016) |
| *Bacillus* phage CAM003 | 25069 | 1.64E+05 | 92% | 0 | 92.06 | 160541 | [NC_024216.1](https://www.ncbi.nlm.nih.gov/nucleotide/NC_024216.1?report=genbank&log$=nucltop&blast_rank=3&RID=VJAMK0N9016) |
| *Bacillus* phage Vinny | 25050 | 1.66E+05 | 92% | 0 | 92.03 | 162865 | [KU737346.1](https://www.ncbi.nlm.nih.gov/nucleotide/KU737346.1?report=genbank&log$=nucltop&blast_rank=4&RID=VJAMK0N9016) |
| *Bacillus* phage Evoli | 25039 | 1.65E+05 | 92% | 0 | 92.02 | 159656 | [NC_024207.1](https://www.ncbi.nlm.nih.gov/nucleotide/NC_024207.1?report=genbank&log$=nucltop&blast_rank=5&RID=VJAMK0N9016) |
| *Bacillus* phage Hoody T | 25034 | 1.65E+05 | 92% | 0 | 92.01 | 159837 | [NC_024205.1](https://www.ncbi.nlm.nih.gov/nucleotide/NC_024205.1?report=genbank&log$=nucltop&blast_rank=6&RID=VJAMK0N9016) |
| *Bacillus* phage Anthony | 32577 | 1.64E+05 | 91% | 0 | 90.96 | 159983 | [MF498901.1](https://www.ncbi.nlm.nih.gov/nucleotide/MF498901.1?report=genbank&log$=nucltop&blast_rank=7&RID=VJAMK0N9016) |
| *Bacillus* phage Janet | 16988 | 1.65E+05 | 91% | 0 | 92.43 | 160705 | [MF288922.1](https://www.ncbi.nlm.nih.gov/nucleotide/MF288922.1?report=genbank&log$=nucltop&blast_rank=8&RID=VJAMK0N9016) |
| *Bacillus* phage Bastille | 18984 | 1.59E+05 | 89% | 0 | 91.87 | 153962 | [NC_018856.1](https://www.ncbi.nlm.nih.gov/nucleotide/NC_018856.1?report=genbank&log$=nucltop&blast_rank=9&RID=VJAMK0N9016) |
| *Bacillus* phage vB_BthM-Goe5 | 25045 | 1.57E+05 | 87% | 0 | 92.03 | 157804 | [MK215646.1](https://www.ncbi.nlm.nih.gov/nucleotide/MK215646.1?report=genbank&log$=nucltop&blast_rank=10&RID=VJAMK0N9016) |
| *Bacillus* phage vB_BcgM | 3192 | 17149 | 20% | 0 | 82.38 | 160441 | [OM743306.1](https://www.ncbi.nlm.nih.gov/nucleotide/OM743306.1?report=genbank&log$=nucltop&blast_rank=11&RID=VJAMK0N9016) |
| *Bacillus* phage SWEP1 | 4503 | 17596 | 19% | 0 | 82.86 | 162461 | [MW787012.1](https://www.ncbi.nlm.nih.gov/nucleotide/MW787012.1?report=genbank&log$=nucltop&blast_rank=12&RID=VJAMK0N9016) |
| *Bacillus* phage B4 | 3675 | 15523 | 19% | 0 | 79.92 | 162596 | [NC_018863.1](https://www.ncbi.nlm.nih.gov/nucleotide/NC_018863.1?report=genbank&log$=nucltop&blast_rank=13&RID=VJAMK0N9016) |
| *Bacillus* phage B5S | 3675 | 15523 | 19% | 0 | 79.92 | 162598 | [NC_047736.1](https://www.ncbi.nlm.nih.gov/nucleotide/NC_047736.1?report=genbank&log$=nucltop&blast_rank=14&RID=VJAMK0N9016) |
| *Bacillus* phage Spock | 3544 | 16315 | 19% | 0 | 79.45 | 164297 | [NC_022763.1](https://www.ncbi.nlm.nih.gov/nucleotide/NC_022763.1?report=genbank&log$=nucltop&blast_rank=15&RID=VJAMK0N9016) |
